# Derivatives of GdAAZTA Conjugated to Amino Acids: A Multinuclear and Multifrequency NMR Study

**DOI:** 10.1021/acs.inorgchem.2c02110

**Published:** 2022-08-09

**Authors:** Daniela Lalli, Ivan Hawala, Marco Ricci, Fabio Carniato, Luca D. D’Andrea, Lorenzo Tei, Mauro Botta

**Affiliations:** † Dipartimento di Scienze e Innovazione Tecnologica, Università del Piemonte Orientale, Viale Teresa Michel 11, 15121 Alessandria, Italy; ‡ Magnetic Resonance Platform (PRISMA-UPO), Università del Piemonte Orientale, Viale Teresa Michel 11, 15121 Alessandria, Italy; § Department of Imaging Chemistry and Biology, School of Biomedical Engineering and Imaging Sciences, King’s College London, Fourth Floor Lambeth Wing, St Thomas’ Hospital London, SE1 7EH, UK; ∥ Istituto di Scienze e Tecnologie Chimiche “G. Natta”, Consiglio Nazionale delle Ricerche, Via M. Bianco 9, 20131 Milano, Italy

## Abstract

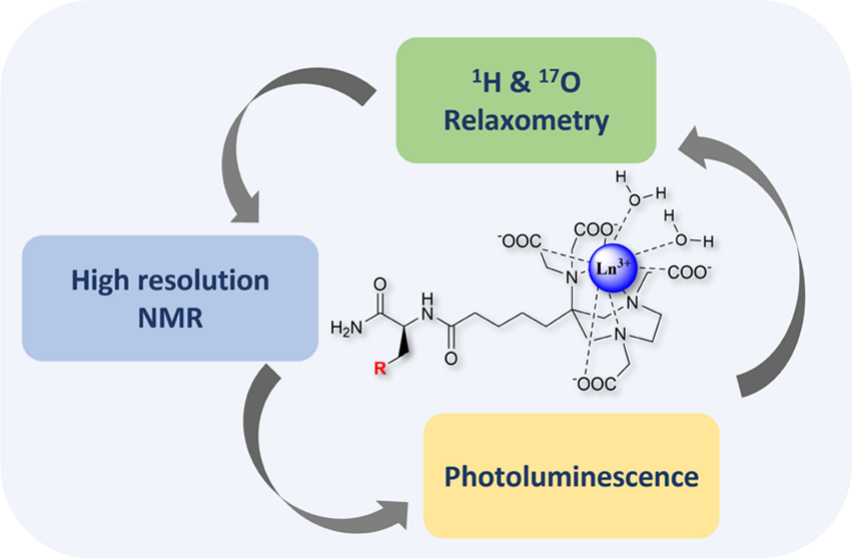

The GdAAZTA (AAZTA = 6-amino-6-methylperhydro-1,4-diazepinetetraacetic acid) complex represents a platform of great interest for the design of innovative MRI probes due to its remarkable magnetic properties, thermodynamic stability, kinetic inertness, and high chemical versatility. Here, we detail the synthesis and characterization of new derivatives functionalized with four amino acids with different molecular weights and charges: l-serine, l-cysteine, l-lysine, and l-glutamic acid. The main reason for conjugating these moieties to the ligand AAZTA is the in-depth study of the chemical properties in aqueous solution of model compounds that mimic complex structures based on polypeptide fragments used in molecular imaging applications. The analysis of the ^1^H NMR spectra of the corresponding Eu(III)-complexes indicates the presence of a single isomeric species in solution, and measurements of the luminescence lifetimes show that functionalization with amino acid residues maintains the hydration state of the parent complex unaltered (*q* = 2). The relaxometric properties of the Gd(III) chelates were analyzed by multinuclear and multifrequency NMR techniques to evaluate the molecular parameters that determine their performance as MRI probes. The relaxivity values of all of the novel chelates are higher than that of GdAAZTA over the entire range of applied magnetic fields because of the slower rotational dynamics. Data obtained in reconstituted human serum indicate the occurrence of weak interactions with the proteins, which result in larger relaxivity values at the typical imaging fields. Finally, all of the new complexes are characterized by excellent chemical stability in biological matrices over time, by the absence of transmetallation processes, or the formation of ternary complexes with oxyanions of biological relevance. In particular, the kinetic stability of the new complexes, measured by monitoring the release of Gd^3+^ in the presence of a large excess of Zn^2+^, is ca. two orders of magnitude higher than that of the clinical MRI contrast agent GdDTPA.

## Abstract 2

Novel GdAAZTA derivatives conjugated to four amino acids were synthesized and characterized through a multi-technique approach. The complexes maintained the favorable thermodynamic and kinetic properties of the parent compound and showed higher relaxivity values in clinical fields. Therefore, they represent a useful model of more complex bio-conjugated structures used in molecular imaging applications.

## Introduction

Magnetic resonance imaging (MRI) represents a powerful tool in diagnostic medicine and preclinical biomedical research that enables visualization of anatomical images with high spatial and temporal resolution in a noninvasive way. The image contrast in MRI depends on the difference in concentration and, primarily, in the relaxation properties of the water proton nuclei present in different body tissues. Although the natural contrast is superb, it is not always sufficient to provide accurate diagnostic information, which can be enhanced with the use of exogenous contrast agents (CAs).^1−3^ These are low-molecular-weight paramagnetic chelates able to improve the contrast-to-noise ratio of MR images by efficiently shortening the relaxation times of nearby water protons. CAs are inorganic probes administered intravenously, which when distributed in the bloodstream extravasate nonspecifically into tissues and are eliminated rapidly through the kidneys. The most used probes in MR diagnostic imaging are Gd-based contrast agents (GBCAs), in which the metal ion reaches its most stable coordination number (CN = 9) by binding octadentate linear or macrocyclic ligands and one water molecule (*q* = 1).^4−6^ Despite the excellent properties characterizing such probes, including high thermodynamic stability, kinetic inertness and rapid clearance, their ability to enhance relaxation is significantly lower than that theoretically predicted. Such issue motivates the search for new classes of CAs with improved efficacy, which can be administered in lower doses, to reduce costs and minimize the possible risks associated with long-term accumulation of Gd^3+^.

Over the last decades, several Gd-complexes with promising features as potential MRI CAs have been developed, among which the 6-amino-6-methylperhydro-1,4-diazepinetetraacetic acid Gd(III)-chelate (GdAAZTA) has stood out for its excellent properties.^7^ In fact, despite being a heptadentate ligand, AAZTA is able to bind Gd(III) with stability comparable to the commercial octadentate analogues. In addition, the two sites available to complete the coordination number nine are occupied by two water molecules (*q* = 2), which impart an increased relaxation enhancement capacity to the chelate.^7^

The efficacy with which a CA relaxes the proton nuclei of nearby water molecules is described by relaxivity (*r*_1_), defined as the increase of the water protons’ longitudinal relaxation rate (*R*_1_) per millimolar unit of concentration of the paramagnetic ion. At clinical magnetic field strengths (1–7 T), *r*_1_ mainly depends on the molecular tumbling rate (τ_R_) of the chelate, hydration state (*q*), the average lifetime (τ_M_) of the coordinated water molecules, and the electronic relaxation parameters (Δ^2^ and τ_V_) of the metal ion.^1,2^ Interestingly, in clinical fields, GdAAZTA shows relaxivity values higher than that measured for clinically used CAs (*r*_1_ = 6.6 mM^–1^ s^–1^ and *r*_1_ ∼5 mM^–1^ s^–1^ at 298 K and 1.5 T, respectively).^8^ Most importantly, unlike many *q* = 2 complexes, GdAAZTA is characterized by high thermodynamic stability and kinetic inertness toward dissociation, transmetallation, and transferrin-mediated demetallation.^9,10^ Another important property of the complex lies in the dynamics of exchange of the two water molecules coordinated to the metal center. We have recently shown that the two inner-sphere waters have substantially different residence lifetimes as a direct consequence of the structural characteristics of the complex.^8^ Such hydration molecules are located at different positions in the coordination polyhedron of the complex, where the one occupying the more sterically hindered capping position exchanges ∼6 times faster than that residing closer to the metal center.^8^

Such favorable properties have promoted the development of several GdAAZTA derivatives with improved relaxivity,^11^ which have found interesting applications in preclinical MRI studies.^12^ In particular, the evidence that high molecular weight, slowly tumbling molecules provide greater *r*_1_ values in the 0.5–1.5 T range of magnetic field strengths has driven the design of several GdAAZTA macromolecular systems, where the chelate is either covalently bound to large substrates or forms noncovalent macromolecular adducts. For instance, substantial efforts have been made in developing (i) dimeric,^13,14^ multimeric, or dendrimeric derivatives alone,^15,16^ or grafted to PEGylated mesoporous silica nanoparticles,^17^ and (ii) lipophilic GdAAZTA complexes capable of assembling in supramolecular aggregates, such as micelles or liposomes,^18^ which can achieve relaxivity values up to 10 times higher than those of the monomeric species. Remarkably, lipophilic GdAAZTA derivatives have also shown the ability to form high-affinity supramolecular complexes with human serum albumin (HSA) if functionalized with suitable aliphatic groups^19,20^ or with bile acid-like side chains.^21^ This confers to the chelate a prolonged lifetime in the bloodstream and high relaxivity properties, which make it a suitable blood pool agent. In addition, GdAAZTA has also been conjugated with a wide range of targeting vectors capable of specific interactions with biomolecules other than HSA, with the aim of developing contrast agents for molecular imaging applications. Such biochemically targeted probes include GdAAZTA coupled with lipids targeting the liver fatty acid binding protein (L-FABP),^22^ with peptidomimetics interacting with integrin α_ν_β_3_ expressed in cancers cells,^23^ and with fibrin targeting peptides,^16,24^ as pathological biomarkers.

Although much effort has been made in developing high-molecular-weight GdAAZTA derivatives with improved relaxometric performances as potential probes for molecular imaging, little is known about the structural changes that could improve their effectiveness. In fact, maximum relaxivity values are not only achievable by slowing down molecular tumbling motions but also by simultaneously fine-tuning the molecular dynamics, exchange dynamics, and electronic parameters, the latter being closely related to the structural features of the metal complex. These bio-conjugated structures are often difficult to characterize in detail with high- and low-resolution NMR techniques because of their intrinsic complexity associated with their high molecular weight and poor solubility.

For these reasons, the incorporation of low-molecular-weight amino acids into the AAZTA ligand, capable of mimicking the influence of polypeptide fragments on the relaxometric properties of more complex structures, could represent a general and effective approach prior to the synthesis of derivatives for molecular imaging applications. It is well established that the introduction of peripheral functionalities in the ligand can affect the molecular parameters that govern the relaxivity of the corresponding Gd(III) chelates. Of course, the conjugation to more complex biomolecules can further change a few relaxometric parameters (e.g., the exchange rate of the metal-bound water molecules and the molecular tumbling rate), but we hypothesize that only the functional groups closest to the metal center are able to influence other key properties of GdAAZTA, such as electronic relaxation and hydration state.

**Scheme 1:**
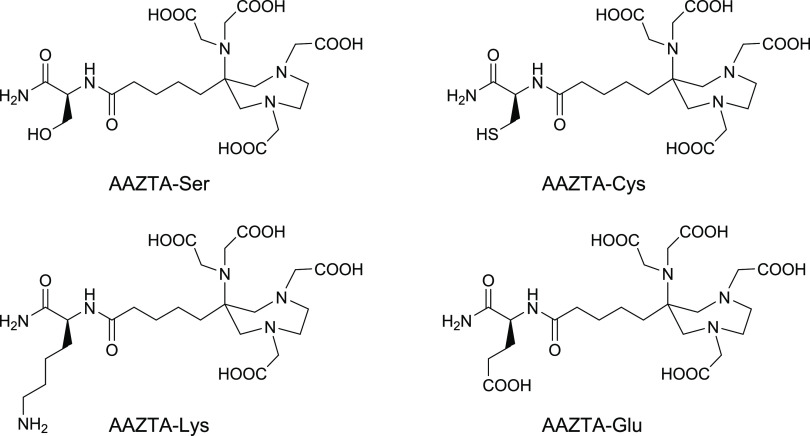
Chemical Structure of the AAZTA-aa Discussed in This Work

Here, we present the synthesis of a novel library of AAZTA-amino acid derivatives (AAZTA-aa) and a comprehensive NMR analysis of the structural and relaxometric properties of their corresponding Gd-chelates. Four AAZTA-aa derivatives were synthesized, each functionalized with residues of different charge, steric hindrance, and hydrophilicity (*i.e.*, l-serine (AAZTA-Ser), l-cysteine (AAZTA-Cys), l-lysine (AAZTA-Lys), and l-glutamic acid (AAZTA-Glu) (Scheme 1)), which are directly linked to the coordination cage of the ligand, exploiting the side chain of the chelator which is not involved in the metal complexation (Scheme 1).

The choice of such systems is based on the evidence that small GBCAs-amino acid conjugates can be accumulated in tumor cells in higher amounts than their benign counterparts, as already demonstrated for GdDOTA-like complexes functionalized with glutamine residues.^25^ To support and complement the information obtained from the relaxometric analysis of the GdAAZTA complexes, ^1^H high-resolution NMR spectra and luminescence lifetime data for the related EuAAZTA complexes were measured.

The results presented in this work offer helpful guidelines for the development of new GdAAZTA derivatives with targeting capabilities and enhanced relaxivity.

## Results and Discussion

### Synthesis of the Ligands and Complexes

A novel library of AAZTA derivatives, where (*t*Bu)_4_-AAZTA-C_4_-COOH was functionalized with four different amino acids (*i.e*., l-serine, l-cysteine, l-lysine, and l-glutamic acid) was successfully obtained, as summarized in Scheme S1. The syntheses were carried out by a solid-phase synthetic approach, following a standardized Fmoc protocol and starting from a Fmoc-Rink Amide MBHA resin. The Fmoc-protected amino acid was separately anchored to the resin using PyBOP (benzotriazol-1-yloxytripyrrolidinophosphonium hexafluorophosphate) as the activating agent and DIPEA (*N*,*N*-diisopropylethylamine) as the base. After removal of the Fmoc protecting group, the chelator was conjugated by reacting overnight (*t*Bu)_4_-AAZTA-C_4_-COOH in the presence of PyBOP and DIPEA. After cleavage from the resin, the final purification was carried out by semi-preparative reversed-phase chromatography, obtaining the final products with a purity of over 95% and an overall yield between 17 and 20% (Figures S1–S4). The solid-phase approach enables a single purification step, avoiding extractions and manual direct-phase chromatography otherwise needed for solution chemistry synthetic procedures. The comparable yields of the four AAZTA-aa derivatives demonstrate the reproducibility of the protocol, which can be generally applied to synthesize different AAZTA-aa conjugates, with high chemical purities, ready to chelate trivalent metal ions used for magnetic resonance applications. The purity of the ligands was confirmed by UPLC-MS analysis (Figures S1–S4) and high-resolution 1D and 2D NMR spectroscopy (Figures S5–S12).

### High-Resolution NMR and Luminescence Studies on EuAAZTA-Glu

To obtain structural information on the paramagnetic GdAAZTA-aa complexes, high-resolution NMR studies were performed on the EuAAZTA derivatives. The ^1^H NMR spectra were acquired at 300 K (Figure S13), showing a single set of resonances similar to what was observed for EuAAZTA.^26^ The ^1^H NMR profiles do not change significantly in the 278–300 K range. This indicates that the introduction of amino acid functionalities does not alter the structure of the AAZTA coordination cage and, most importantly, suggests the presence of a single isomer in solution.

To assess the hydration state (*q*) of the GdAAZTA-aa complexes in solution, luminescence measurements were carried out on the EuAAZTA-Glu chelate and compared with that of the EuAAZTA complex (Figure S14). Luminescence decay profiles were measured over time on the two Eu(III)-complexes dissolved in pure H_2_O and in D_2_O under excitation at 370 nm (Figure S14). The so-obtained decay curves were fitted by a single exponential function to obtain the fluorescence lifetimes.^27^ Both complexes share a bis-hydrated coordination sphere (*q* = 2) as calculated by comparing the fluorescence lifetimes measured in H_2_O and D_2_O (Table S1).^28^ We assume that all of the GdAAZTA-aa family members have the same number of inner-sphere water molecules, since they share an identical coordination cage.

### pH Dependency of Proton Relaxivity

The chemical stability of the GdAAZTA-aa complexes was evaluated by measuring the ^1^H relaxivity (*r*_1_) as a function of pH. The *r*_1_ values were recorded on ∼2 mM aqueous solutions of the GdAAZTA-aa complexes in the pH range between 2.0 and 11.0 (Figure S15) at 32 MHz and 298 K. The experimental results show constant values of *r*_1_ over the entire range of pH investigated, indicating excellent chemical stability of the metal complexes and an unchanged hydration state. It is particularly significant that *r*_1_ does not decrease at basic pH, as commonly observed in *q* = 2 Gd-complexes, as this indicates the lack of formation of ternary complexes with the dissolved carbonate, which involves the displacement of the metal-bound water molecules.^29,30^ Furthermore, we can safely conclude that the protonation state of the residues does not affect the pH dependency of relaxivity.

### ^1^H NMRD Profiles

To obtain information on the molecular and dynamic parameters responsible for the relaxation properties of GdAAZTA-aa derivatives, a detailed analysis of the field-dependent relaxivity profiles was performed.

**Figure 1:**
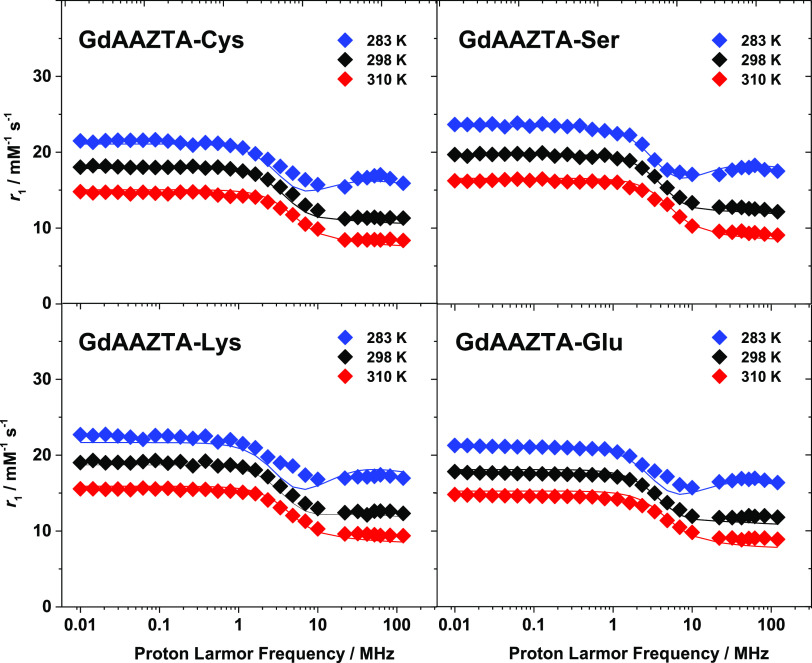
^1^H Nuclear magnetic relaxation dispersion profiles recorded on the GdAAZTA-aa complexes at pH 7.0 at different temperatures of 283 K, 298 K, and 310 K. The solid lines represent the fits of the data, according to the details provided in the text.

Nuclear magnetic relaxation dispersion (NMRD) profiles were acquired on ∼2 mM aqueous solutions of the GdAAZTA-Cys, GdAAZTA-Ser, GdAAZTA-Lys, and GdAAZTA-Glu complexes at neutral pH, by measuring relaxivity (*r*_1_) in the ^1^H Larmor frequency range between 0.01 and 120 MHz and at three different temperatures (283, 298, and 310 K) (Figure 1). The profiles were analyzed by Solomon–Bloembergen–Morgan^31−34^ and Freed^35^ equations that describe the inner- and the outer-sphere contributions to the relaxation, respectively (see ESI for more details on the equations used). The NMRD profiles of the chelates display very similar behavior to each other and to GdAAZTA, which is typical of low-molecular-weight systems with fast molecular tumbling rates.^36^ The profiles are characterized by a low-field plateau between 0.01 and 1 MHz, followed by a single dispersion at about 4 MHz, and a high-field plateau between 20 and 120 MHz. For all of the GdAAZTA-aa derivatives, the relaxivity values measured over the entire Larmor frequency range decrease with increasing temperature, as expected for small-sized complexes characterized by fast exchange regimes (*T*_1M_ > τ_M_). Under these conditions, *T*_1M_ (∼μs) is longer than the mean residency time of the metal-bound water molecule (τ_M_ ∼ 100 ns) and limits the proton relaxivity, influencing its temperature dependence. In turn, *T*_1M_ depends on the correlation time (τ_c_), which is largely dominated by the rotational dynamics (τ_R_ ∼ ps) for small complexes. Consequently, at rising temperatures, the increased rotational dynamics (shorter τ_R_ and τ_c_) causes an increase in *T*_1M_ and, therefore, a decrease in relaxivity. Such a trend is clearly visible in the variable temperature (VT) profiles of *r*_1_ acquired on the GdAAZTA-aa chelates, in the temperature range between 283 and 320 K, at 32 MHz (Figure S16). In the VT-NMR profiles, *r*_1_ decreases with increasing temperatures following a mono-exponential decay, as expected for systems in fast exchange conditions. However, the analysis of these curves does not allow obtaining quantitative information on the exchange dynamics that characterizes the two inner-sphere water molecules. In fact, in the fast exchange regimes, τ_M_ does not influence *r*_1_, which is dominated by *T*_1M_, instead. In such conditions, *r*_1_ largely depends on *q* and τ_R_, as a first approximation. Therefore, the 2-fold relaxivity enhancement measured at 32 MHz for the GdAAZTA-aa complexes with respect to the GdAAZTA chelate is attributable to the lengthening of τ_R_ associated with an increase of their molecular weight (MW). As expected, a linear correlation of relaxivity on the molecular size is observed by plotting *r*_1_ vs MW for the GdAAZTA-aa derivatives, with the exception of GdAAZTA-Ser, which slightly deviates from linearity (Figure S17).^2^ Such interesting behavior can be attributed to the occurrence of second sphere water molecules hydrating the polar hydroxyl group of serine, which contribute to the relaxivity in a non-negligible way (*r*_1_^SS^). Likewise, the rotational correlation time displays the same linear dependence with the molecular weight, thus allowing a rough estimate of the rotational dynamics (τ_R_ ∼100–150 ps) (Figure S17).^2^

### ^17^O NMR Measurements

**Figure 2:**
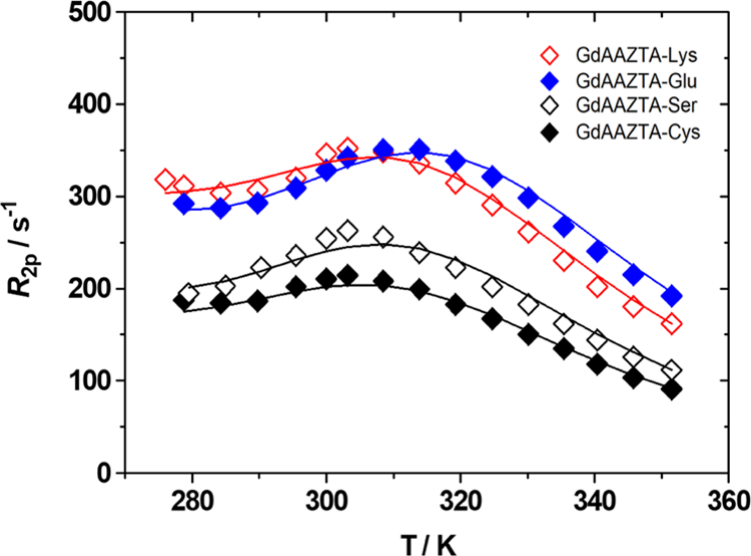
^17^O-reduced transverse relaxation rate as a function of temperature acquired on the GdAAZTA-aa complexes: (aa = Cys, Ser, Lys, and Glu), at 11.74 T. The solid lines represent the fits of the data, as described in the text.

Detailed information on the inner-sphere water exchange dynamics of the GdAAZTA-aa complexes was obtained by measuring the ^17^O transverse relaxation rates (*R*_2_ = 1/*T*_2_) and chemical shift (Δω_r_) of the bulk water as a function of temperature (280–350 K) at high field (11.74 T) (Figures 2 and S18). The ^17^O-*R*_2_ profiles of the four GdAAZTA-aa complexes show similar asymmetrical “bell” shapes characterized by maxima around 300 K and unusual trends at temperatures below 285 K, resembling that observed for GdAAZTA.^8^

Such behavior is attributable to the presence of two Gd-bound water molecules occupying different positions of the coordination polyhedron with different bond lengths and therefore characterized by different rates of exchange. The hydration water closer to the metal center is in an intermediate exchange regime with the bulk and generates a ^17^O-*R*_2_ maximum at ∼300 K. The more labile and distant from Gd(III) water ligand has a faster rate of exchange (*k*_ex_ = 1/τ_M_), and is responsible for the ^17^O-*R*_2_ increase observed below 285 K.^8^ As an example, the distinct contribution of the two water molecules to the ^17^O-*R*_2_ profile of GdAAZTA-Glu is emphasized in Figure S19.

In principle, the presence of multiple isomers in solution featuring different water exchange rates could also account for such an unusual trend of the ^17^O-*R*_2_ profiles, as already observed for other Gd(III) chelates.^37,38^ However, such hypothesis is ruled out by the presence of a single set of signals in the variable temperature NMR spectra of the EuAAZTA-aa complexes (Figure S13).^26^

### Quantitative Analysis of the ^1^H NMRD and ^17^O NMR Data

A global analysis of the experimental ^1^H NMRD and ^17^O NMR data was performed simultaneously. Solomon–Bloembergen–Morgan^31−34^ and Freed^35^ equations that describe the inner- and the outer-sphere contributions to the relaxation were used for the analysis of the NMRD profiles. Swift–Connick equations were used for the analysis of the ^17^O NMR data.^39^ The equations have been modified to account for the different contributions of the two water ligands, which are characterized by different residence lifetimes (τ_M1_ and τ_M2_), associated exchange enthalpies (Δ*H*_M1_ and Δ*H*_M2_), and hyperfine scalar coupling constants (*A*_O_/ℏ_1_ and *A*_O_/ℏ_2_).

To aid the fitting procedure, some of the structural and dynamic parameters affecting ^1^H *r*_1_ and ^17^O-*R*_2_ data were set to the values reported for the GdAAZTA complex.^8^

In particular, the number of water ligands (*q* = 2, according to the photoluminescence analyses reported above), the distance between the inner-sphere water protons and Gd(III) (*r* = 3.0 Å), the closest distance between an outer-sphere water molecule and the paramagnetic center (*a* = 4.0 Å), the relative diffusion coefficient of the outer-sphere water molecules and the complex at 298 K (^298^*D* = 2.24 × 10^5^ cm^2^ s^–1^), the activation energy for the diffusion coefficient (*E*_D_ = 20 kJ mol^–1^), and the activation energy of zero-field splitting modulation (*E*_v_ = 1.0 kJ mol^–1^) were taken into account.

An excellent fit of the ^1^H NMRD and ^17^O NMR data of the GdAAZTA-aa complexes (Figures 1 and 2) was obtained with the parameters listed in Tables 1 and S2. The parameters describing the electron spin relaxation of the paramagnetic metal (*i.e*., the correlation time associated with the modulation of the zero-field splitting (ZFS) interaction τ_V_ and the square of the mean ZFS energy Δ^2^) assume values that are similar among the different GdAAZTA-aa complexes and in agreement with those reported for GdAAZTA.^8^ This indicates that AAZTA functionalization with amino acids of different nature does not alter the symmetry of the coordination cage and, therefore, the electronic relaxation properties of the metal ion in the Gd-complex.

**Table 1:** Parameters Obtained from the Simultaneous Analysis of ^17^O NMR and ^1^H NMRD Data Acquired on the GdAAZTA-aa Complexes

	GdAAZTA-Ser	GdAAZTA-Cys	GdAAZTA-Lys	GdAAZTA-Glu	GdAAZTA^b^
^298^*r*_1_ (60 MHz) (mM^–1^ s^–1^)	12.4	11.2	12.5	11.9	6.2
Δ^2^/10^19^ s^–2^	2.56	2.30	2.40	2.30	2.6
τ_V_/ps	30.0	31.0	30.0	30.0	30.0
τ_M1_/ns	23.3	24.8	29.8	22.5	29
τ_M2_/ns	206	190	219.2	245	169
Δ*H*_M1_/*k*J mol^–1^	18.0	18.5	20.0	19.0	20.0
Δ*H*_M2_/*k*J mol^–1^	30.9	29.0	29.8	29.2	29.5
τ_R_/ps	115	121	140	127	74
*q*	2^a^	2^a^	2^a^	2^a^	2^a^

a Parameter fixed during the fitting procedure.

b Data from ref (8).

On the other hand, variations in the exchange dynamics of the two metal-bound water molecules are observable among the different GdAAZTA-aa derivatives and with respect to GdAAZTA. In particular, the water exchange dynamics is modulated by the steric hindrance and charge of the amino acid side chains. In fact, the substitution of the methyl group with more sterically hindered amino acid functionalities reduces the residence time of the more labile water ligand with respect to GdAAZTA, while increasing the residence lifetime of that closer to Gd(III), as observed for the negatively charged GdAAZTA-aa complexes (τ_M1_ (GdAAZTA-aa) = 22.5–24.8 ns and τ_M1_ (GdAAZTA) = 29 ns, τ_M2_ (GdAAZTA-aa) = 190–245 ns and τ_M2_ (GdAAZTA) = 169 ns) (Tables 1 and S2). Such a trend can be attributed to the increased steric crowding at the water binding site that favors the dissociation of the more labile water molecule and consequently stabilizes the less hindered one, as already observed for the negatively charged LnAAZTA complexes. For the latter systems, the lanthanide contraction along the series increases the steric compression and leads to the loss of the more labile water molecule between Ho and Er. This phenomenon is associated with a remarkable stabilization of the residual water ligand, whose water exchange rate drops by 2–3 orders of magnitude toward the end of the series.^26^ On the other hand, in the case of the neutrally charged GdAAZTA-Lys complex, the τ_M1_ reduction and τ_M2_ elongation expected from the increased steric crowding are mitigated by more favorable water–metal interactions compared to the negatively charged complexes (Table 1). In fact, the neutral charge of the chelate promotes stronger electrostatic water–metal interactions and causes a slowdown of the overall water exchange kinetics. The residency time of the rapidly exchanging water ligand is higher than that found for the negatively charged complexes and comparable to that of GdAAZTA. Moreover, the dynamics of the slower exchanging water ligand is reduced with respect to GdAAZTA and to the negatively charged complexes, with the exception of GdAAZTA-Glu (Table 1). Surprisingly, the GdAAZTA-Glu complex is characterized by the coordinated water molecule residing for the longest time on the metal center despite its bulkier and negatively charged side chain.

For all of the GdAAZTA-aa complexes, the hydration equilibrium of the more labile water molecule has an enthalpy value ∼1.5 times lower than that associated with the other (Δ*H*_M1_ ∼ 20 kJ mol^–1^ and Δ*H*_M2_ ∼ 30 kJ mol^–1^), as also observed for GdAAZTA.^8^

The values of the rotational correlation times obtained from the data fits are sensibly longer than that of GdAAZTA (τ_R_ = 115-140 ps vs τ_R_ = 74 ps, respectively), as expected for complexes with increased size and molecular mass. The decrease of the rotational dynamics of the GdAAZTA-aa chelates mainly accounts for their remarkable relaxivity enhancement compared to GdAAZTA. The τ_R_ value calculated for GdAAZTA-Cys allows excluding a possible dimerization of the complex under these experimental conditions, promoted by the oxidation of the -SH group. Interestingly, the highest relaxivity value is measured for GdAAZTA-Ser chelate (*r*_1_ = 12.4 mM^–1^ s^–1^, at 60 MHz, 298 K) despite having the lowest molecular mass. This can be attributed to possible hydrogen bonds between the polar OH-group of the serine side chain and water molecules, which generate a second sphere contribution to the relaxation that was considered during the data analysis. The experimental data are in excellent agreement with the presence of a single second sphere water molecule (*q*^SS^ = 1) at a distance of 3.5 Å from the paramagnetic center (Table S2). The so-obtained best-fitting curves correctly describe the experimental data. The electronic parameters (Δ^2^ = 2.3–2.6 × 10^19^ s^–2^; τ_V_ = 30–31 ps) and the hyperfine coupling constants obtained by the fit procedure for the four analyzed complexes (Tables 1 and S2) are in excellent agreement with those reported in previous studies, pointing out that this type of functionalization keeps the coordination cage of the metal ion unchanged.

### Kinetic Inertness of the GdAAZTA-aa Complexes

**Figure 3:**
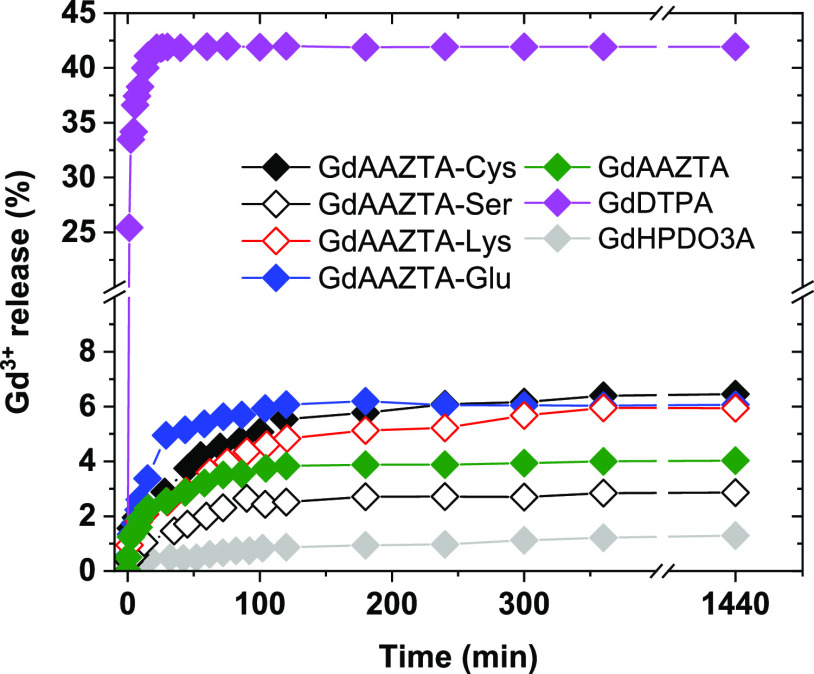
Gd^3+^ release (%) as a function of time measured for the GdAAZTA-aa complexes: (aa = Cys, Ser, Lys, and Glu), and for GdAAZTA ([Gd^3+^] = 1 mM, [Zn^2+^] = 25 mM, pH 6.30, 310 K, 10 MHz). Gd^3+^ release for GdHPDO3A and GdDTPA are also reported for comparison.

An important feature of GdAAZTA is the good kinetic inertia, a very relevant aspect for bioimaging applications. To obtain a qualitative evaluation of the inertness of the GdAAZTA-aa derivatives toward the transmetallation processes, the rates of metal ion displacement of the chelates were determined by challenging the complexes with 25 equiv of Zn^2+^ (pH 6.30, 310 K) and monitoring the Gd^3+^ release by recording *T*_1_ values at 10 MHz as a function of time (Figure 3). Such a test is widely adopted in the literature for a preliminary evaluation of the inertness of the MRI probes *in vitro*.^40^ In fact, as the concentration of Zn^2+^ in the blood is relatively high (55–125 mM), it may favor the displacement of a significant amount of Gd^3+^. Other potential competitors *in vivo*, including Cu^2+^, Ca^2+^, or Fe^3+^, can be neglected, as Cu^2+^ concentration is low (1–10 mM); Ca^2+^ ions typically show a low affinity constant with organic ligands, and Fe^3+^ is not easily available for transmetallation.^41^

Furthermore, the same measurements were performed on the GdAAZTA, GdHPDO3A, and GdDTPA complexes as a comparative benchmark. The pseudo-first-order constants of the transmetallation reaction, obtained from the fitting of the experimental data (Experimental section), are reported in Table 2. The kinetic constants found for the GdAAZTA-aa complexes are comparable with those of GdAAZTA, indicating that the ligand functionalization with amino acid residues does not alter the kinetic stability of the Gd(III)-chelate. In addition, the excellent kinetic stability of the GdAAZTA-aa complexes is comparable to (GdHPDO3A) or *ca.* 2 order of magnitude higher (GdDTPA) than that of clinically approved MRI contrast agents.^40^

**Table 2:** Pseudo-First-Order Kinetic Constants for the Gd^3+^ Transmetallation ([Gd^3+^] = 1 mM, [Zn^2+^] = 25 mM, pH = 6.30, 310 K, 10 MHz) Obtained from the Fitting Procedure for the Series of Complexes Examined

complexes	*K*_obs_ (s^–1^)
GdAAZTA	(5.9 ± 0.9) × 10^–4^
GdAAZTA-Ser	(3.5 ± 0.3) × 10^–4^
GdAAZTA-Cys	(2.6 ± 0.3) × 10^–4^
GdAAZTA-Lys	(2.1 ± 0.3) × 10^–4^
GdAAZTA-Glu	(8.1 ± 0.9) × 10^–4^
GdDTPA	(1.1 ± 0.1) × 10^–2^
GdHPDO3A	(1.2 ± 0.2) × 10^–4^

### Stability of the GdAAZTA-aa Complexes in Biological Matrices

**Figure 4:**
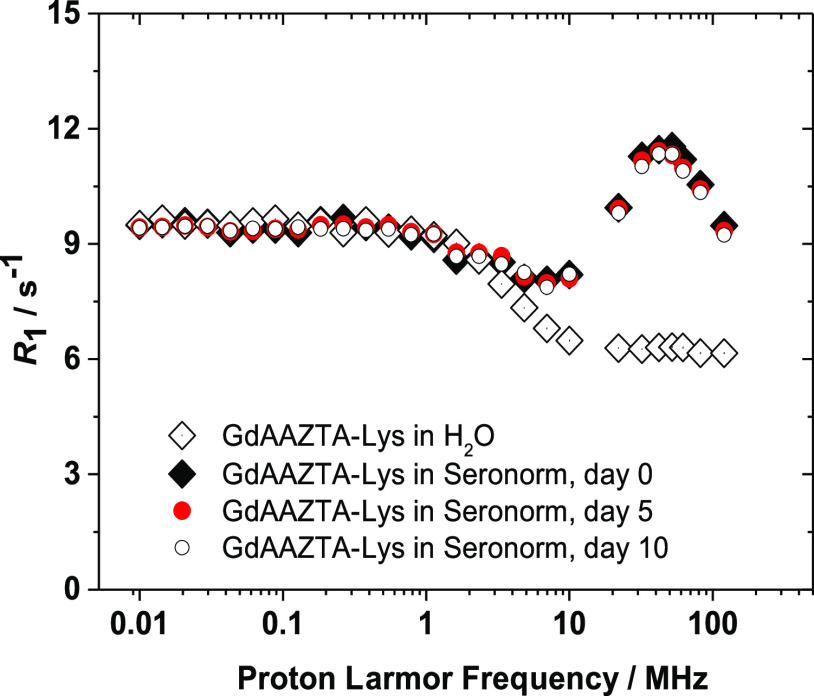
Comparison of *R*_1_ values as a function of the magnetic field strength (0.01–120 MHz) of the GdAAZTA-Lys complex in pure water (◊) and in the presence of Seronorm, (298 K, pH 7.4, [Gd^3+^] = 0.5 mM) over time.

*In vitro* stability studies of the GdAAZTA-aa complexes in a simulated physiological environment were carried out to evaluate their stability for *in vivo* applications. The lyophilized human serum Seronorm was used to simulate the biological matrix. ^1^H NMR relaxometric studies were carried out on aqueous solutions of the GdAAZTA-aa complexes in the presence of reconstituted human serum (Figures 4 and S19) over 10 days to check the chemical stability of the complexes (Figure 4). In addition, ^1^H NMRD profiles acquired on the GdAAZTA-aa complexes in pure water and in Seronorm were compared to assess the occurrence of possible interactions between the paramagnetic complexes and the biological matrix (Figures 4 and S20).

The profiles show shape and amplitude that are significantly different in the frequency range between 10 MHz and 120 MHz and nearly identical at lower frequency values. Unlike GdAAZTA, the GdAAZTA-aa chelates show a broad hump in the high-fields range in the profiles collected in the presence of Seronorm, which is absent in pure water. For all GdAAZTA derivatives, the *R*_1_ peak in the NMRD profiles in Seronorm represents a notable increase, corresponding to an enhancement of 45.3, 22.6, and 39.7% (32 MHz) for GdAAZTA-Cys, GdAAZTA-Ser, and GdAAZTA-Glu, respectively, and as much as 84.2% for GdAAZTA-Lys (52 MHz). Considering the high stability characterizing the GdAAZTA-aa complexes, such behavior suggests the occurrence of relatively strong interactions between the chelates and the serum proteins, with the formation of high molecular weight supramolecular adducts. Such slowly rotating systems are characterized by reduced rotational dynamic (lengthening of τ_R_), which causes the observed relaxivity increases, particularly pronounced for GdAAZTA-Lys. At lower magnetic field strengths, instead, the relaxivity values of the complexes in Seronorm remain unaltered, indicating that the electronic relaxation parameters, dominating at low fields, do not change appreciably upon interactions in the serum matrix. Finally, the NMRD profiles acquired after 5 and 10 days do not show significant differences compared to the profile acquired at time zero. This indicates the absence of metal ion release phenomena and/or the formation of ternary complexes with biological oxyanions, which further highlights the remarkable chemical stability of these complexes. Therefore, the collected data support the suitability for *in vivo* preclinical applications of these novel GdAAZTA-aa complexes.

## Conclusions

In conclusion, the herein synthetic procedure can be exploited to obtain different amino acid-chelator conjugates with high chemical purity, ready for complexation with gadolinium or other trivalent metal ions of interest in magnetic resonance applications (*e.g.,* paraCEST). The use of a solid-phase synthesis approach enables the performance of just one purification step, avoiding extractions and manual direct-phase chromatography, as expected by solution chemistry. The synthesis of four different AAZTA-amino acid derivatives with comparable overall yields demonstrates the reproducibility of the synthetic protocol, which can be generally applied for the generation of different chelator-amino acid conjugates. In-depth NMR analyses were performed on the AAZTA-aa ligands and their Gd(III) and Eu(III) complexes to understand their structural, relaxometric, and stability properties. High-resolution NMR studies showed the presence of a single isomer in solution and highlighted structural analogy with the EuAAZTA complex, indicating the retention of the structural properties of the coordination cage upon introduction of amino acid functionalities. The hydration number of the GdAAZTA-aa complexes is also retained, as demonstrated by luminescence lifetimes data. The two coordinated water molecules exhibit significantly different exchange rates, where the one closer to the metal resides for a longer time at the metal center, while the other exchanges up to 10 times faster. This behavior, which is consistent with that recently reported for the GdAAZTA complex, has been accessed by the simultaneous analysis of the water ^1^H longitudinal and ^17^O transverse relaxation rates and chemical shift variations as a function of the magnetic field strength and temperature, respectively. The resulting picture shows that, for negatively charged complexes, the increased steric compression on the coordination cage accelerates the exchange dynamics of the more labile water molecule while slowing down the dynamics of that more tightly bound to the metal center with respect to GdAAZTA. Such behavior is amplified the higher the steric hindrance and the negative charge are, as observed for GdAATZA-Glu that possesses the highest exchange rate for the most labile water molecule and the lowest for the least labile one. However, for the neutrally charged complex GdAAZTA-Lys, the same effect caused by increased steric compression is mitigated by the more favorable electrostatic interactions between the Gd(III) and the water ligands. In addition, from a detailed analysis of the molecular parameters controlling the relaxation properties of GdAAZTA-aa derivatives, it emerges that their increased size proportionally reduces their rotational dynamics in solution, thus increasing their relaxivity values in clinical fields. GdAAZTA-Ser represents the only exception, showing a significant second sphere contribution to relaxation. In the case of our new complexes, the incorporation of the amino acid groups in the ligand structure does not alter the hydration state and the electronic parameters of the parent GdAAZTA. This is an important information because it allows for making reliable predictions on the relaxivity of more complex bio-conjugated structures.

The kinetic and chemical stability of the GdAAZTA-aa complexes were preliminarily examined to evaluate their potential applicability for *in vivo* studies. The kinetic inertness of the GdAAZTA-aa complexes is comparable to that of GdAAZTA and to the macrocyclic clinical contrast agent GdHPDO3A. Finally, the GdAAZTA derivatives show excellent chemical stability in biological matrices over time, with the absence of metal ion release phenomena or the formation of ternary complexes with oxyanions of biological relevance. In light of these results, we can conclude that the GdAAZTA-aa complexes can be considered suitable for preclinical MRI studies.

## Materials and Methods

All Fmoc (Fluorenylmethyloxycarbonyl)-protected amino acids, Fmoc-Rink Amide MBHA resin, and PyBOP were purchased from Novabiochem (Darmstad, Germany), Sigma-Aldrich (Darmstad, Germany) and Iris Biotech (Marktredwitz, Germany). All other reagents were purchased from Sigma-Aldrich (Darmstad, Germany). All solvents were purchased from VWR International (Radnor, USA) and were used without further purification. 6-[Bis[2-(1,1-dimethylethoxy)-2-oxoethyl]amino]-6-(5-carboxypentyl)tetrahydro-1H-1,4-diazepine-1,4(5H)-Diacetic acid *N,N*′-bis(1,1-dimethylethyl)ester ((*t*Bu)_4_-AAZTA-C_4_-COOH) was synthesized in accordance with Manzoni et al. protocol.^23^

The RP-HPLC preparative purifications were carried out on a Waters AutoPurification system (3100 Mass Detector 600 Quaternary Pump Gradient Module, 2767 Sample Manager, and 2487 UV/Visible Detector), employing an Atlantis Prep. D C18OBD, 5 μm, 19 × 100mm column. UPLC-MS analyses were performed using a Waters ACQUITY UPLC H-Class coupled with an ESI source, a quadrupole (QDa) mass analyzer, and a dual-wavelength UV/Vis TUV Detector, employing an ACQUITY UPLC Peptide BEH C18 column (300 Å, 1.7 μm, 2.1 × 100 mm). ^1^H and ^13^C NMR spectra of the ligands and their complexes with europium were recorded at 298 K on a Bruker AVANCE 500 spectrometer.

### Synthesis of the AAZTA-aa Ligands

The synthetic procedures used for the preparation of the ligands are summarized in Scheme S1. Briefly, 400 mg of a Fmoc-Rink Amide MBHA resin (loading 0.59 mmol/g) were swelled for 10 min with DMF (*N*, *N*-Dimethylformamide). All reaction steps were performed under gentle stirring (35 rpm) and at room temperature. The resin was filtered, and 10 mL of a solution of piperidine 20% in DMF was added to the reactor vessel. After 30 min, the resin was filtered and washed with DMF. Typically, 5 equiv of Fmoc-AA-OH (AA = Ser, Cys, Lys, and Glu), 10 equiv of DIPEA (*N*,*N*-diisopropylethylamine), and 4.5 equiv of PyBOP previously dissolved in DMF (10 mL) were added to the reactor vessel. After 2 h, the resin was filtered and extensively washed with DMF. The resin was filtered, and 10 mL of capping solution (Acetic Anhydride/DIPEA/DMF 1:1:3) was added to the reactor vessel. After 30 min, the resin was filtered and extensively washed with DMF. The resin was filtered, and 10 mL of a solution of piperidine 20% in DMF was added to the reactor vessel. After 30 min, the resin was filtered and washed with DMF. Then, 1.5 equiv of (*t*Bu)_4_-AAZTA-C_4_-COOH, 3 equiv of DIPEA, and 1.35 equiv of PyBOP previously dissolved in DMF (10 mL) were added to the reactor vessel. The reaction was left to stir overnight. The resin was filtered and extensively washed with DMF, DCM (methylene chloride), and diethyl ether. Then, 10 mL of cleavage solution (Trifluoroacetic Acid/Triisopropylsilane/H_2_O 95:2.5:2.5) was added to the reaction vessel, and the reaction was stirred overnight. The crude product was precipitated in cold diethyl ether, and the final purification was achieved by semi-preparative RP-HPLC on a Waters AutoPurification system. Eluent: (A) 0.1% TFA in H_2_O, (B) 0.1% TFA in CH_3_CN. Gradient profile; linear gradient from 2 to 20% of B in 7 min, linear gradient from 20 to 100% in 3 min, isocratic at 100% for 1 min. Flow rate; 15 mL/min. The pure product was isolated as a homogeneous peak with a retention time of ca. 4 minutes. The solvent was evaporated in vacuo, and the product was lyophilized from water to give the desired product as a white solid. The purity of the final product was checked by analytical UPLC-MS. Eluent: (A) 0.05% TFA in H_2_O, (B) 0.05% TFA in CH_3_CN. Gradient profile; linear gradient from 5 to 50% of B in 7 min, linear gradient from 50 to 100% in 3 min, isocratic at 100% for 3 min; flow rate of 0.4 mL/min and UV detection at 210 nm.

#### 2,2′-((6-(5-((1-Amino-3-hydroxy-1-oxopropan-2-yl)amino)-5-oxopentyl)-1,4-bis(carboxymethyl)-1,4-diazepan-6-yl)azanediyl)diacetic acid (**AAZTA-Ser**)

**AAZTA-Ser** was synthesized, purified, and characterized following the generic synthetic procedure. *p* = 22.04 mg, 18%. RT: 1.08 min. Purity 99%. The ^1^H and ^13^C NMR resonance assignment refers to the numbering of the ligand atoms reported in Figures S5–6: δ^H^ 4.38 (t, *J* = 5.3 Hz, 1H, 14), 3.84 – 3.79 (m, 6H, 10-11-15), 3.75 (s, 4H, 12-13), 3.72 – 3.66 (m, 2H, 3′–4′), 3.52 – 3.43 (m, 6H, 1-2-3″–4″), 2.31 (t, *J* = 7.2 Hz, 2H, 9), 1.59–1.52 (m, 4H, 6-8), 1.29 (m, 2H, 7). δ^C^ 176.9 (2C, a–b), 175.7 (2C, c–d), 170.8 (2C, e–f), 63.5 (C, 5), 61.2 (1C, 15), 59.1 (2C, 10–11), 58.9 (2C, 12–13), 55.3 (1C, 14), 53.2 (2C, 1–2), 52.2 (2C, 3-4), 34.9 (1C, 9), 33.4 (1C, 6), 25.3 (1C, 8), 22.1 (1C, 7). ESI-MS (*m*/*z*): calcd For C_21_H_35_N_5_O_11_ (M + H)^+^ 534.54; found, 534.41.

#### 2,2′-((6-(5-((1-Amino-3-mercapto-1-oxopropan-2-yl)amino)-5-oxopentyl)-1,4-bis(carboxymethyl)-1,4-diazepan-6-yl)azanediyl)diacetic acid (**AAZTA-Cys**)

**AAZTA-Cys** was synthesized, purified, and characterized following the generic synthetic procedure. *p* = 25.55 mg, 20%. RT: 1.18 min. Purity 96%. The ^1^H and ^13^C NMR resonance assignment refers to the numbering of the ligand atoms reported in Figures S7–S8: δ^H^ 4.44 (dd, *J* = 8.0, 4.9 Hz, 1H, 14), 3.79 (s, 4H, 10–11), 3.75 (s, 4H, 12–13), 3.71–3.66 (m, 2H, 3′-4′), 3.52–3.50 (m, 2H, 3″–4″), 3.49 (m, 4H, 1–2), 2.93 (dd, *J* = 14.1, 5.0 Hz, 1H, 15′), 2.83 (dd, *J* = 14.1, 8.05 Hz, 1H, 15″), 2.32 (t, *J* = 7.2 Hz, 2H, 9), 1.58–1.54 (m, 4H, 6–8), 1.30 (m, 7). δ^C^ 177.0 (2C, a–b), 175.4 (2C, c–d), 170.9 (2C, e–f), 63.7 (C, 5), 59.0 (4C, 10–11–12–13), 58.9 (1C, 15), 55.4 (1C, 14), 53.4 (2C, 1–2), 52.1 (2C, 3-4), 34.9 (1C, 9), 33.3 (1C, 6), 25.4 (1C, 8), 22.1 (1C, 7). ESI-MS (*m*/*z*): calcd For C_21_H_35_N_5_O_10_S (M + H)^+^ 550.60; found, 550.21.

#### 2,2′-((1,4-Bis(carboxymethyl)-6-(5-((1,6-diamino-1-oxohexan-2-yl)amino)-5-oxopentyl)-1,4-diazepan-6-yl)azanediyl)diacetic acid (**AAZTA-Lys)**

**AAZTA-Lys** was synthesized, purified, and characterized following the generic synthetic procedure. *p* = 23.62 mg, 17%. RT: 0.83 min. Purity 99%. The ^1^H and ^13^C NMR resonance assignment refers to the numbering of the ligand atoms reported in Figures S9–S10: δ^H^ 4.21 (dd, *J* = 8.7, 5.4 Hz, 1H, 14), 3.82 (s, 4H, 10–11), 3.74 (s, 4H, 12–13), 3.71–3.67 (m, 2H, 3′–4′), 3.51–3.48 (m, 2H, 3″–4″), 3.48 (s, 4H, 1–2), 2.95 (t, *J* = 7.8 Hz, 2H, 18), 2.27 (t, *J* = 7.4 Hz, 2H, 9), 1.82–1.75 (m, 1H, 15′), 1.73–1.68 (m, 1H, 15″), 1.65 (m, 2H, 17), 1.56–1.52 (m, 4H, 6–8), 1.42 (m, 2H, 16), 1.27 (m, 2H, 7). δ^C^ 176.8 (2C, a-b), 175.8 (2C, c–d), 170.8 (2C, e–f), 63.3 (C, 5), 59.3 (2C, 10–11), 59.0 (2C, 12–13), 53.3 (1C, 14), 53.1 (2C, 1–2), 52.2 (2C, 3–4), 39.2 (1C, 18), 34.9 (1C, 9), 33.6 (1C, 6), 30.4 (1C, 15), 26.3 (1C, 17), 25.3 (1C, 8), 22.2 (2C, 7-16). ESI-MS (*m*/*z*): calcd For C_24_H_42_N_6_O_10_ (M + H)^+^ 575.63; found, 575.31.

#### 2,2′-((6-(5-((1-Amino-4-carboxy-1-oxobutan-2-yl)amino)-5-oxopentyl)-1,4-bis(carboxymethyl)-1,4-diazepan-6-yl)azanediyl)diacetic acid (**AAZTA-Glu**)

**AAZTA-Glu** was synthesized, purified, and characterized following the generic synthetic procedure. *p* = 24.18 mg, 18%. RT: 0.88 min. Purity 97%. The ^1^H and ^13^C NMR resonance assignment refers to the numbering of the ligand atoms reported in Figures S11,12: δ^H^ 4.27 (dd, *J* = 9.5, 5.2 Hz, 1H, 14), 3.83 (s, 4H, 10–11), 3.70 (s, 4H, 12–13), 3.66–3.60 (m, 2H, 4′–3′), 3.46–3.42 (m, 2H, 4″–3″), 3.42 (s, 4H, 1–2), 2.44–2.40 (m, 2H, 16), 2.24 (t, *J* = 7.2 Hz, 2H, 9), 2.10–2.03 (m, 1H, 15′), 1.93–1.86 (m, 1H, 15″), 1.53–1.46 (m, 4H, 6-8), 1.25–1.19 (m, 2H, 7). δ^C^ 176.9 (1C, b), 176.7 (1C, a), 176.2 (1C, g), 176.1 (2C, c–d), 170.7 (2C, f–e), 62.9 (1C, 5), 59.6 (2C, 3–4), 58.7 (2C, 12–13), 52.7 (1C, 14), 52.6 (2C, 1–2), 52.4 (2C, 10–11), 35.0 (1C, 9), 33.8 (1C, 6), 30.1 (1C, 16), 26.2 (1C, 15), 25.4 (1C, 8), 22.1 (1C, 7). ESI-MS (*m*/*z*): calcd For C_23_H_37_N_5_O_12_ (M + H)^+^ 576.57; found, 576.31.

### Preparation of the LnAAZTA-aa Complexes

LnAAZTA-aa complexes were prepared by adding 1.1 equiv of LnCl_3_ salts to an aqueous solution of the AAZTA-aa ligands at pH = 5.5. After the addition, the pH was adjusted to 6.0 with an aqueous solution of NaOH 1 M, and the solution was stirred at room temperature (r.t.) for 12 h. Then, the pH was increased to 10 with 0.1 M NaOH, and the solution was stirred for 3 h to promote the precipitation of the uncomplexed Ln(III) as insoluble hydroxides. The solution was centrifuged (10,000 rpm, 5 min, r.t.); the supernatant was filtered through 0.2 μm filters and neutralized with dilute HCl to separate Ln hydroxides from the solution. The concentration of Ln(III) complexes was evaluated by ^1^H NMR measurements at 11.7 Tesla, using the well-established bulk magnetic susceptibility method.^42^

GdAAZTA-Ser: ESI-MS (*m*/*z*): calcd For GdC_21_H_31_N_5_O_11_ (M + H)^+^ 687.69; found, 687.34.

GdAAZTA-Cys: ESI-MS (*m*/*z*): calcd For GdC_21_H_31_N_5_O_10_S (M + H)^+^ 703.75; found, 703.37.

GdAAZTA-Lys: ESI-MS (*m*/*z*): calcd For GdC_24_H_38_N_6_O_10_ (M + H)^+^ 728.78; found, 728.51.

GdAAZTA-Glu: ESI-MS (*m*/*z*): calcd For GdC_23_H_33_N_5_O_12_ (M + H)^+^ 729.72 found, 729.72.

EuAAZTA-Cys: ^1^H NMR δ^H^: 8.5 (br s, 4H), 6.3 (s, 2H), 5.2 (br s, 2H), 3.9–3.2 (br s, 8H), 2.5 (br s, 2H), −1.9 (br s, 2H), −7.5 (br s, 2H), −9.3 (br s, 2H).

EuAAZTA-Ser: ^1^H NMR δ^H^: 8.9 (br s, 2H), 8.1 (br s, 2H), 6.1 (s, 2H), 5.0 (br s, 2H), 4.2 (br, 2H), 3.7–3.1 (br s, 6H), 2.7 (br s, 2H), −1.9 (br s, 2H), −7.5 (br s, 2H), −9.5 (br s, 2H).

EuAAZTA-Lys: ^1^H NMR δ^H^: 8.8 (br s, 2H), 8.2 (br s, 2H), 6.1 (s, 2H), 5.1 (br s, 2H), 4.3 (b, 2H), 4.0–3.0 (br s, 8H), 2.8 (br s, 2H), 2.1 (br, 2H), 1.9 (br, 4H), −1.9 (br s, 2H), −7.6 (br s, 2H), −9.5 (br s, 2H).

EuAAZTA-Glu: ^1^H NMR δ^H^: 8.3 (br s, 2H), 8.6 (br s, 2H), 6.2 (s, 2H), 5.1 (br s, 2H), 4.9 (br, 1H), 4.6 (br s, 2H), 3.7 (br s, 2H), 3.6 (br s, 2H), 3.3 (br s, 2H), 3.0–2.0 (br s, 6H), −2.1 (br s, 2H), −7.8 (br s, 2H), −9.5 (br s, 2H).

### ^1^H NMRD Measurements

1/*T*_1_ 1H Nuclear magnetic relaxation dispersion (NMRD) profiles were acquired with a fast field cycling (FFC) Stelar SMARtracer relaxometer (Stelar s.r.l., Mede, PV, Italy) over a range of proton Larmor frequencies from 9.97 × 10^–3^ to 10 MHz, with an uncertainty from 1/*T*_1_ of ca. 1%. Data in the range 20–120 MHz proton Larmor frequency were measured with a high-field relaxometer (Stelar) equipped with the HTS-110 3T Metrology cryogen-free superconducting magnet. The NMRD profiles were acquired at three different temperatures (283, 298, and 310 K). The temperature was controlled during the measurements with a Stelar VTC-91 heater airflow equipped with a copper–constantan thermocouple (uncertainty of ±0.1 °C). The real temperature inside the probe was monitored by a Fluke 52k/j digital thermometer (Fluke, Zürich, Switzerland). The data were collected using the standard inversion recovery sequence (16 experiments, 3 scans) with a typical 90° pulse width of 3.5 μs. The reproducibility of the data was within ±0.5%.

Relaxivity measurements of aqueous solutions of the GdAAZTA-aa complexes were performed to gain information on the stability of the Gd^3+^ complexes. *r*_1_ values were measured for the 2 mM solutions of the complexes, at 32 MHz, 298 K, in the pH range ∼2.0–10.0. *r*_1_ values remain constant in the entire pH range. The pH dependence was measured by raising the pH from 6.5 to basic values with the addition of 0.1 M NaOH and then by lowering it to acid values with the addiction of 0.1 M HCl.

^1^H NMRD profiles were acquired on GdAAZTA-aa complexes dissolved in reconstituted human serum (Seronorm) for the stability studies ([Gd^3+^] = 0.5 mM, pH = 7.4 and 298 K).

### ^17^O NMR *T*_2_ Measurements

Variable temperature ^17^O NMR measurements were recorded on a Bruker Avance III spectrometer (11.7 T) equipped with a 5 mm double resonance Z-gradient broadband probe and Bruker BVT-3000 unit for temperature control. The sample was prepared in a 3 mm NMR tube by mixing 188 μl of a ∼10–20 mM complex solution at physiological pH, 22 μL of D_2_O with 10% of tert-butanol, and 10 μL of H_2_^17^O (Cambridge Isotope, 2% isotope enrichment). The transverse relaxation rates were calculated from the signal full width at a half-height. The bulk magnetic susceptibility contribution was subtracted from the ^17^O NMR shift data using the ^1^H NMR shifts of the tert-butanol signal as the internal reference. Other details of the instrumentation, experimental methods, and data analysis have been previously reported.^43^

^1^H NMR measurements. The one-dimensional ^1^H and ^13^C Nuclear magnetic resonance (NMR) spectra of the AAZTA-aa ligands and of the EuAAZTA-Glu complex in solution were recorded at 298 K with a Bruker Avance III spectrometer equipped with a wide bore 11.7 Tesla magnet. Briefly, 5–10 mM solutions were prepared by dissolving the samples in isotopically enriched water (D_2_O) for NMR analyses. The 2D COSY spectra acquired on the ligands were collected using a standard phase-insensitive COSY sequence with gradient coherence selection, with 2048 acquired data points in F2, 256 times increments in F1, 16 scans, a 2 s recycle delay, and a spectral window (both F2 and F1) of 10 ppm.

### Zn(II) Transmetallation Kinetics

Displacement of Gd(III) by Zn(II) was measured by monitoring the evolution of free Gd(III) concentration by *T*_1_-relaxometry. Solutions of the Gd(III) complexes and ZnCl_2_·6H_2_O were prepared in a 1:25 molar ratio at pH 6.3. The *r*_1_ data were fitted to the below equation^44^
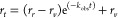
Where *r*_r_, *r*_v_, and *r*_t_ are respectively initial relaxivity, at equilibrium, and at time *t* of the transmetallation reaction, and *K*_obs_ is the pseudo-first-order kinetic constant of the transmetallation reaction.

### Luminescence Measurements

Two solutions of 1 mM EuAAZTA-Glu chelate were prepared by dissolving the complex in 1 mL of H_2_O and D_2_O, respectively. Luminescence decays were measured on a Horiba Jobin Yvon Model IBH FL-322 Fluorolog 3 spectrofluorometer working in the time-correlated single-photon counting mode (TCSPC) using a SpectraLED (370 nm) for the excitation and monitoring the emission signal of Eu(III) at 615 nm. The signals were collected with an IBH DataStation Hub photon counting module, and the data were analyzed with the DAS6 (Horiba Jobin Yvon IBH) software. The obtained decay curves were then fitted with a mono-exponential decay function to obtain the lifetime of the excited level in H_2_O and D_2_O. The hydration state of the complex was then determined by applying Parker and co-workers’ equation.^30^

## Supporting Information Available

UPLC-MS and high-resolution NMR spectra of the AAZTA-aa ligands. ^1^H NMR spectra of EuAAZTA-aa. Photoluminescence intensity decay curves of EuAAZTA and EuAAZTA-Glu samples. pH and temperature dependence of relaxivity and ^17^O NMR data of GdAAZTA-aa chelates. NMRD profiles of GdAAZTA-aa in Seronorm matrix (PDF)
