# Supplementary material for: Derivatives of GdAAZTA Conjugated to Amino Acids: A Multinuclear and Multifrequency NMR Study
Source: Inorg Chem. 2022 Aug 9;61(33):13199–209. doi: 10.1021/acs.inorgchem.2c02110 (PMC9400103; doi:10.1021/acs.inorgchem.2c02110)
Supplement: Supplementary file 1 — ic2c02110_si_001.pdf [file ic2c02110_si_001.pdf]

SUPPLEMENTARY INFORMATION

Derivatives of GdAAZTA conjugated to amino acids: a multinuclear and multifrequency NMR study

Daniela Lalli,[†‡] *Ivan Hawala,*[#] *Marco Ricci,*[†] *Fabio Carniato,*[†‡] *Luca D. D'Andrea,*[§]* *Lorenzo Tei,*[†] *Mauro Botta*[†‡]*

† Dipartimento di Scienze e Innovazione Tecnologica, Università del Piemonte Orientale, Viale T. Michel 11, 15121 Alessandria, Italy. mauro.botta@uniupo.it

‡ Magnetic Resonance Platform (PRISMA-UPO), Università del Piemonte Orientale, Viale T. Michel 11, 15121 Alessandria, Italy.

Department of Imaging Chemistry and Biology, School of Biomedical Engineering and Imaging Sciences, King's College London, Fourth Floor Lambeth Wing, St Thomas' Hospital London, SE1 7EH (UK)

§ Istituto di Scienze e Tecnologie Chimiche "G. Natta", Consiglio Nazionale delle Ricerche, Via M. Bianco 9, 20131 Milano (Italy). lucadomenico.dandrea@cnr.it

Summary

Scheme S1: Reaction scheme for the synthesis of AAZTA-aa derivatives.

Figure S1: UPLC-MS analysis of pure AAZTA-Cys.

Figure S2: UPLC-MS analysis of pure AAZTA-Glu.

Figure S3: UPLC-MS analysis of pure AAZTA-Lys.

Figure S4: UPLC-MS analysis of pure AAZTA-Ser.

Figure S5: 1D ^{1}H and ^{13}C NMR spectra acquired for the AAZTA-Cys ligand.

Figure S6: 2D ^{1}H-^{1}H COSY 2D NMR spectrum acquired for the AAZTA-Cys ligand.

Figure S7: 1D ^{1}H and ^{13}C NMR spectra acquired for the AAZTA-Ser ligand.

Figure S8: 2D ^{1}H-^{1}H COSY 2D NMR spectrum acquired for the AAZTA-Ser ligand.

Figure S9: 1D ^{1}H and ^{13}C NMR spectra acquired for the AAZTA-Lys ligand.

Figure S10: 2D ^{1}H-^{1}H COSY 2D NMR spectrum acquired for the AAZTA-Lys ligand.

Figure S11: 1D ^{1}H and ^{13}C NMR spectra acquired for the AAZTA-Glu ligand.

Figure S12: 2D ^{1}H-^{1}H COSY 2D NMR spectrum acquired for the AAZTA-Glu ligand.

Figure S13: ^{1}H NMR spectra acquired for the EuAAZTA-aa complexes at 300 K.

Figure S14: Photoluminescence intensity decay profiles over time for the EuAAZTA and EuAAZTA-Glu complexes in H_2O e D_2O.

Figure S15: pH dependence of r_1 measured for aqueous solutions of the GdAAZTA-aa complexes at 298 K and 32 MHz.

Figure S16: Temperature dependence of r_1 measured for aqueous solutions of the GdAAZTA-aa complexes at 32 MHz.

Figure S17: Left: plot of r_1 (0.5 T and 298 K) for aqueous solutions of GdAAZTA derivatives and other bi-hydrated Gd(III) chelates versus molecular weight. Right: Plot of the corresponding rotational correlation times as a function of molecular weight.

Figure S18: Reduced transverse ^{17}O relaxation rates and chemical shifts measured at 11.74 T for the GdAAZTA-aa complexes.

Figure S19: ^{17}O-R_2 profile of GdAAZTA-Glu.

Figure S20: Comparison of R_1 values (0.01-120 MHz) measured on the GdAAZTA-aa complexes in pure water and Seronorm® matrix.

Table S1: Excited state lifetime values in H_2O and in D_2O for the EuAAZTA and EuAAZTA-Glu complexes obtained from the fitting procedure of the PL data.

Table S2. Parameters obtained from the simultaneous analysis of ^{17}O NMR and ^{1}H NMRD data acquired on the GdAAZTA-aa complexes.

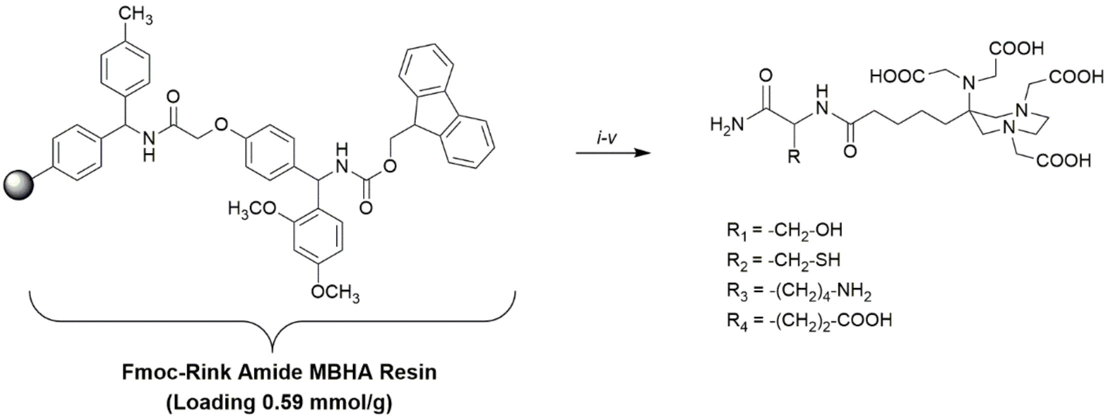

Scheme S1. Reaction scheme for the synthesis of AAZTA-aa derivatives: *i*) piperidine 20%, 30 min; *ii*) Fmoc-NH-CH-$R_{1\text{-}4}$-COOH, PyBOP, DIPEA, 2h; *iii*) piperidine 20%, 30 min; *iv*) $(tBu)_4$-AAZTA-C_4-COOH, PyBOP, DIPEA, 24 h; *v*) TFA/TIS/H_2O 95:2.5:2.5, 24 h.

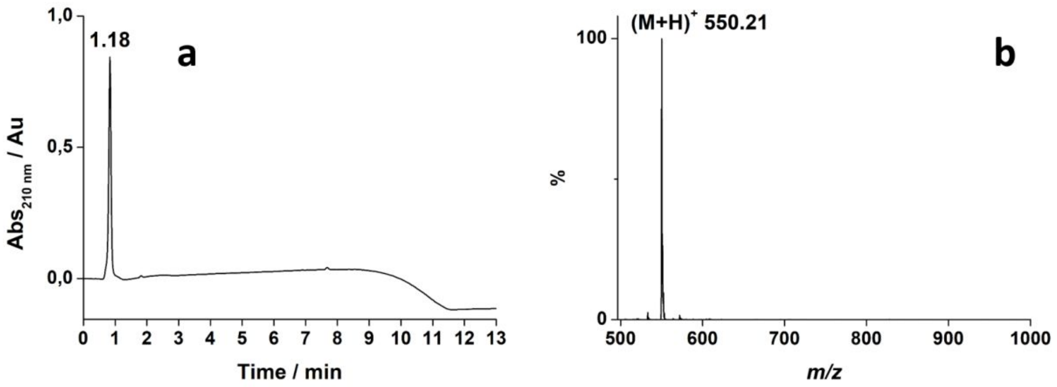

Figure S1. UPLC-MS analysis of pure AAZTA-Cys: a) chromatographic profile revealed at 210 nm and ESI-MS spectrum (b) of the peak at 1.28 min retention time.

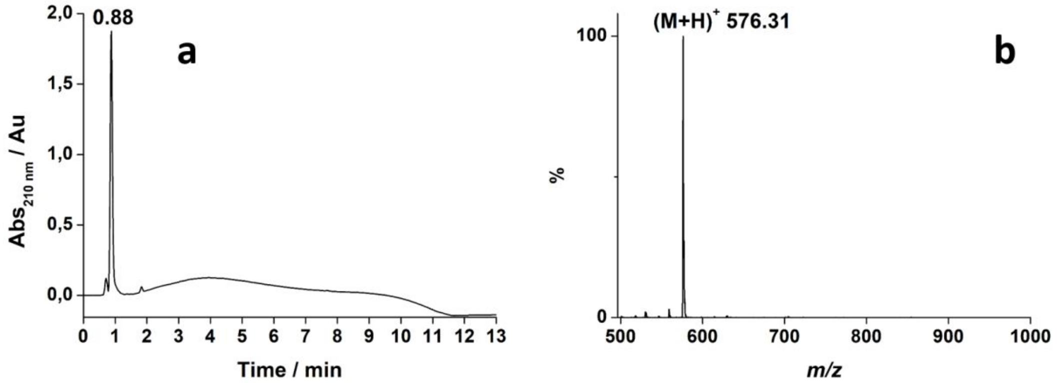

Figure S2. UPLC-MS analysis of pure AAZTA-Glu: a) chromatographic profile revealed at 210 nm and ESI-MS spectrum (b) of the peak at 0.88 min retention time.

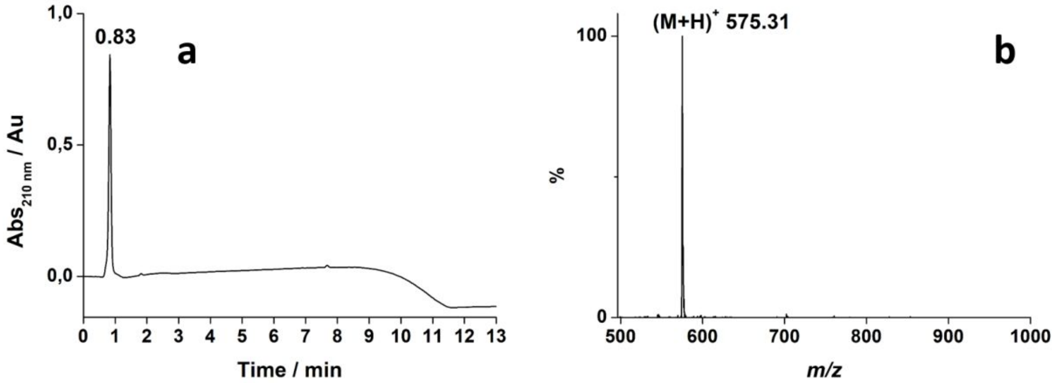

Figure S3. UPLC-MS analysis of pure AAZTA-Lys: a) chromatographic profile revealed at 210 nm and ESI-MS spectrum (b) of the peak at 0.83 min retention time.

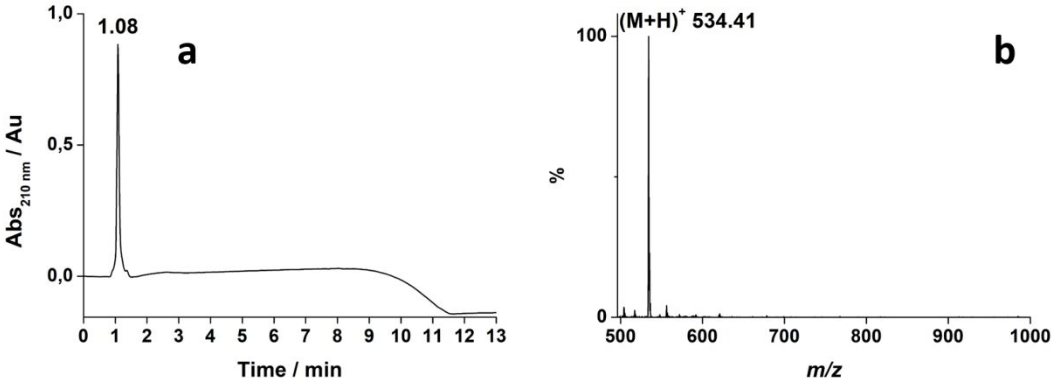

Figure S4. UPLC-MS analysis of pure AAZTA-Ser: a) chromatographic profile revealed at 210 nm and ESI-MS spectrum (b) of the peak at 1.08 min retention time.

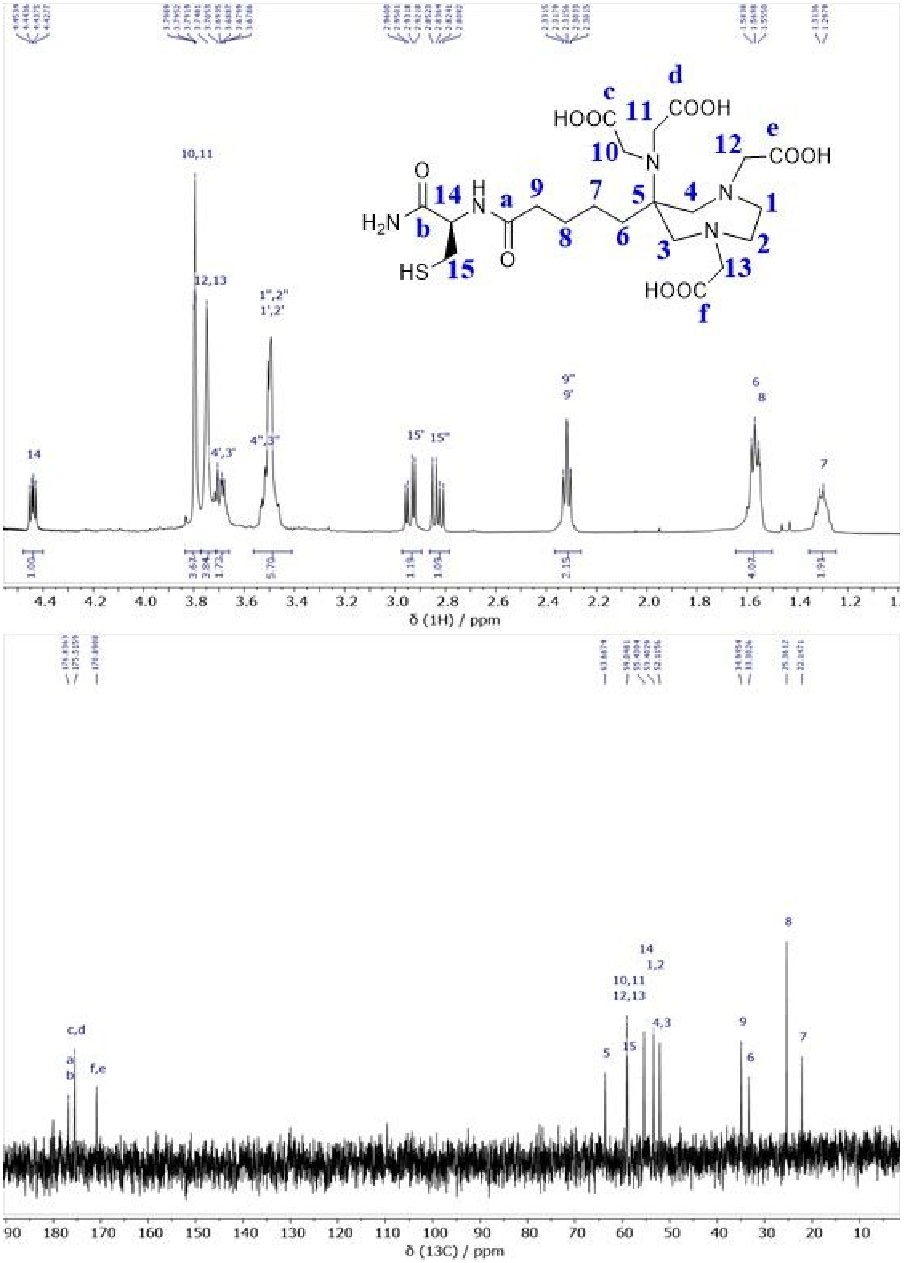

Figure S5. 1D ^{1}H and ^{13}C NMR spectra acquired for the AAZTA-Cys ligand at 300 K, 11.7 Tesla.

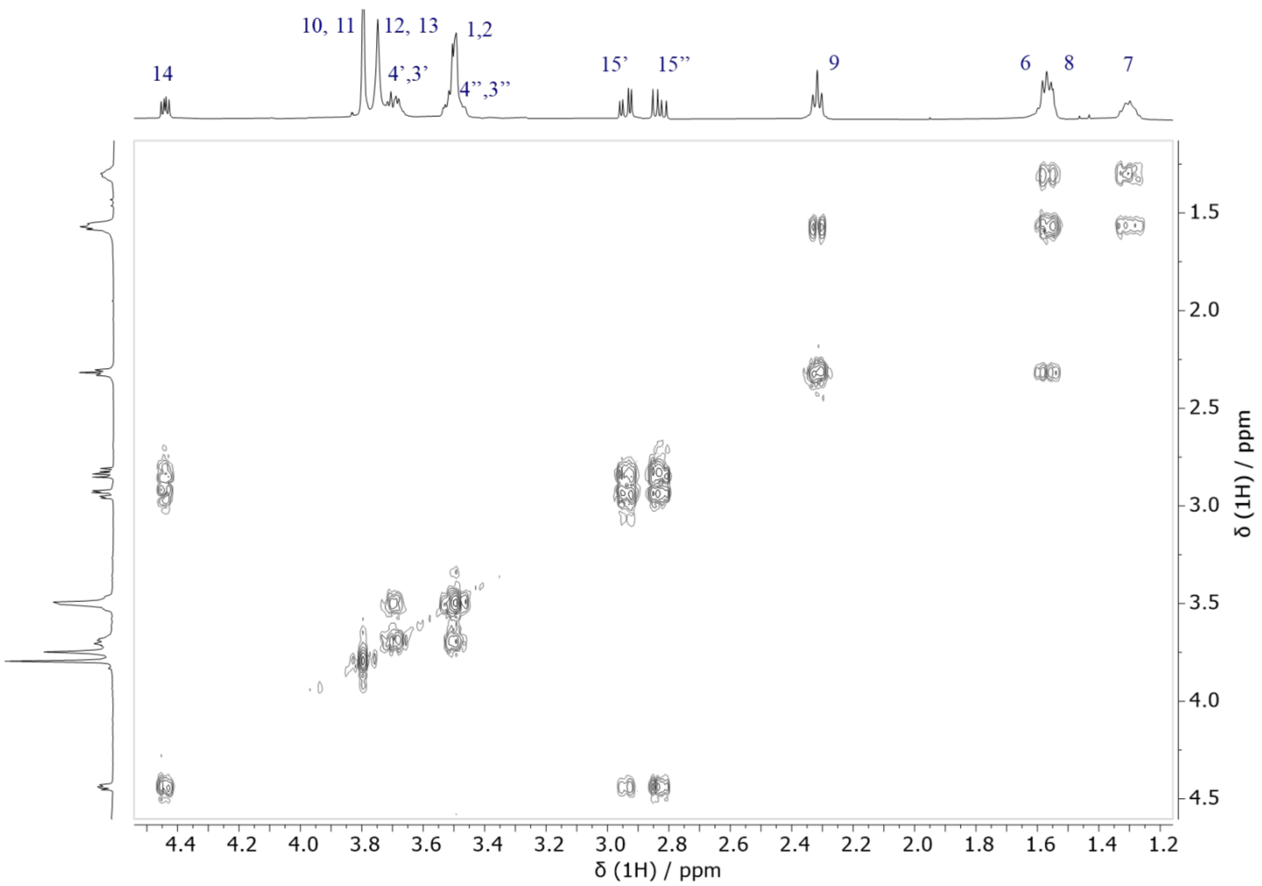

Figure S6. ^{1}H-^{1}H COSY 2D NMR spectrum acquired for the AAZTA-Cys ligand at 300 K, 11.7 Tesla.

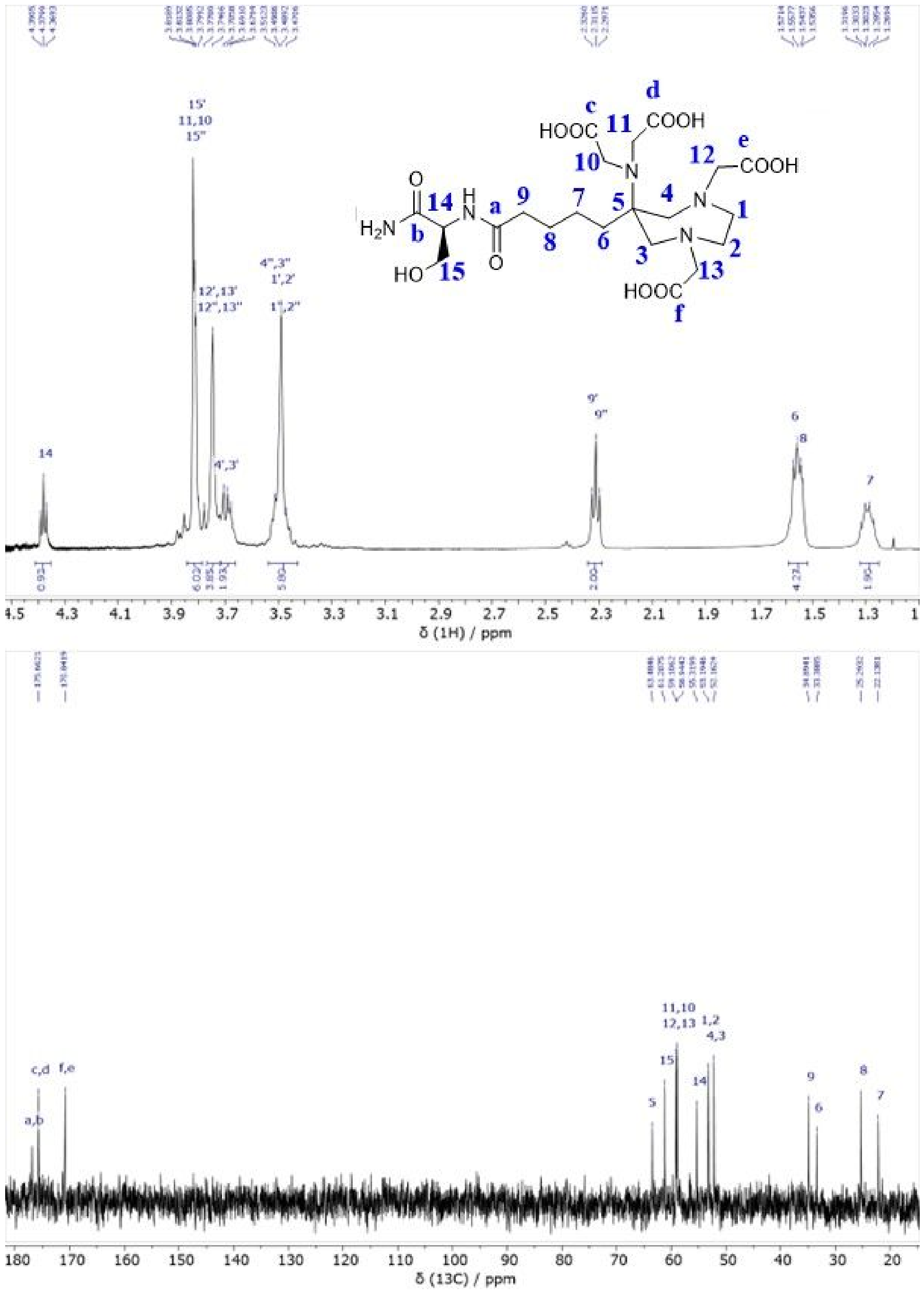

Figure S7. 1D ^{1}H and ^{13}C NMR spectra acquired for the AAZTA-Ser ligand at 300 K, 11.7 Tesla.

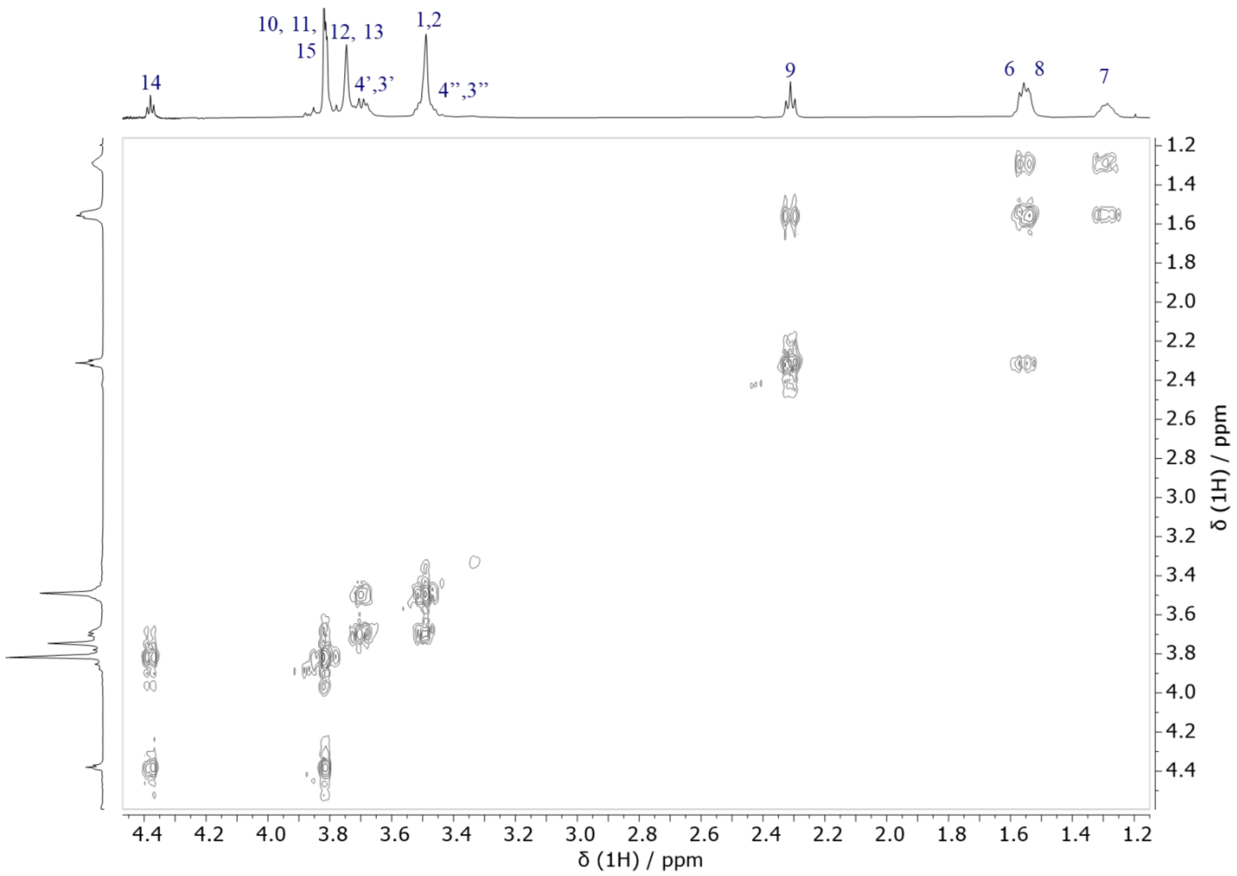

Figure S8. ^1H-^1H COSY 2D NMR spectrum acquired for the AAZTA-Ser ligand at 300 K, 11.7 Tesla.

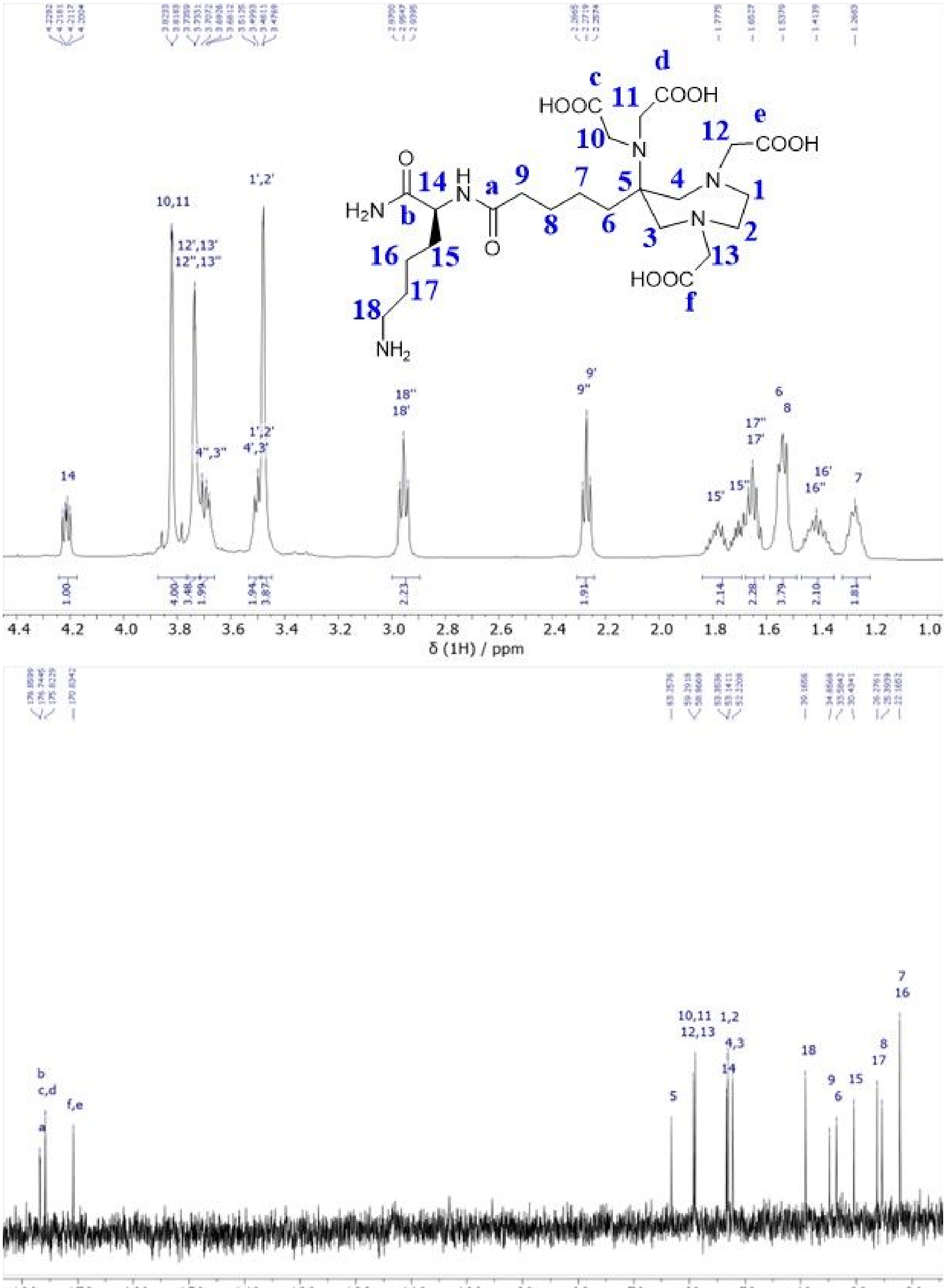

Figure S9. 1D ^{1}H and ^{13}C NMR spectra acquired for the AAZTA-Lys ligand at 300 K, 11.7 Tesla.

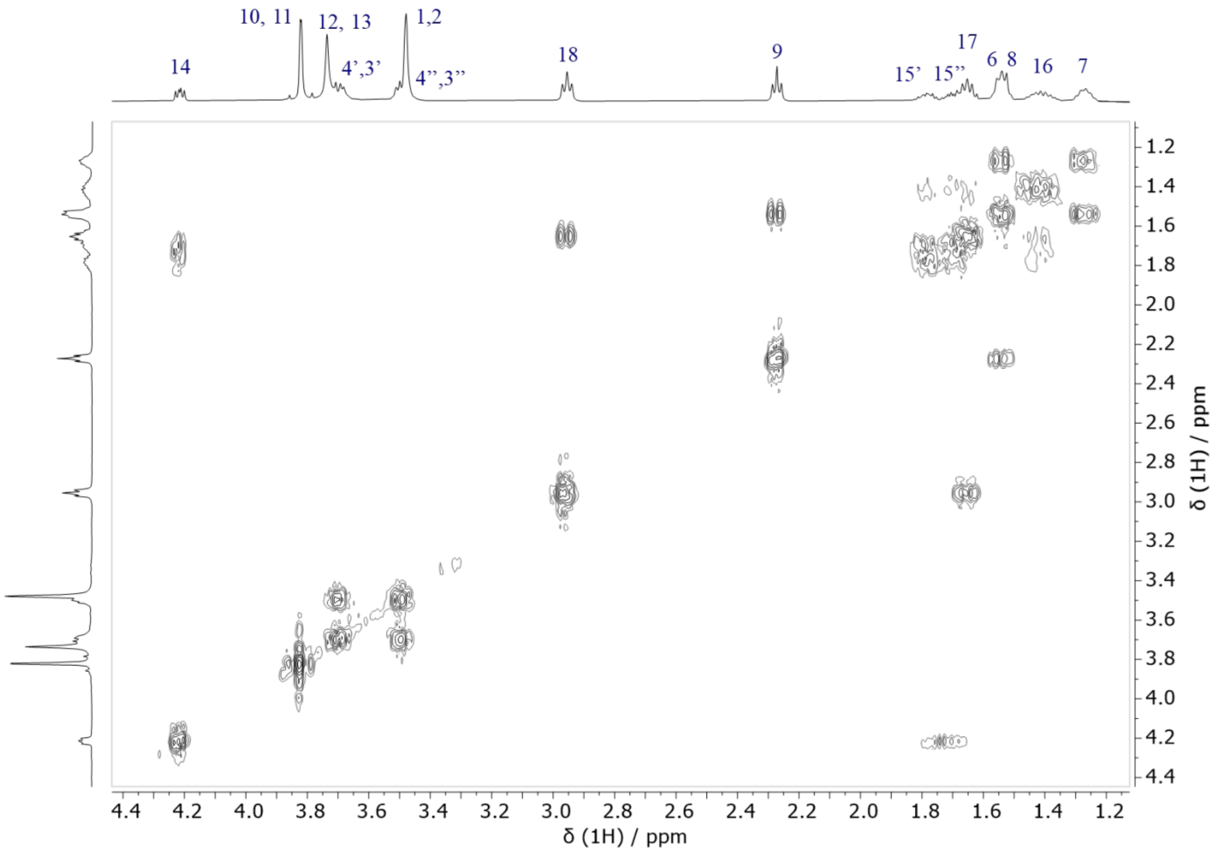

Figure S10. ^{1}H-^{1}H COSY 2D NMR spectrum acquired for the AAZTA-Lys ligand at 300 K, 11.7 Tesla.

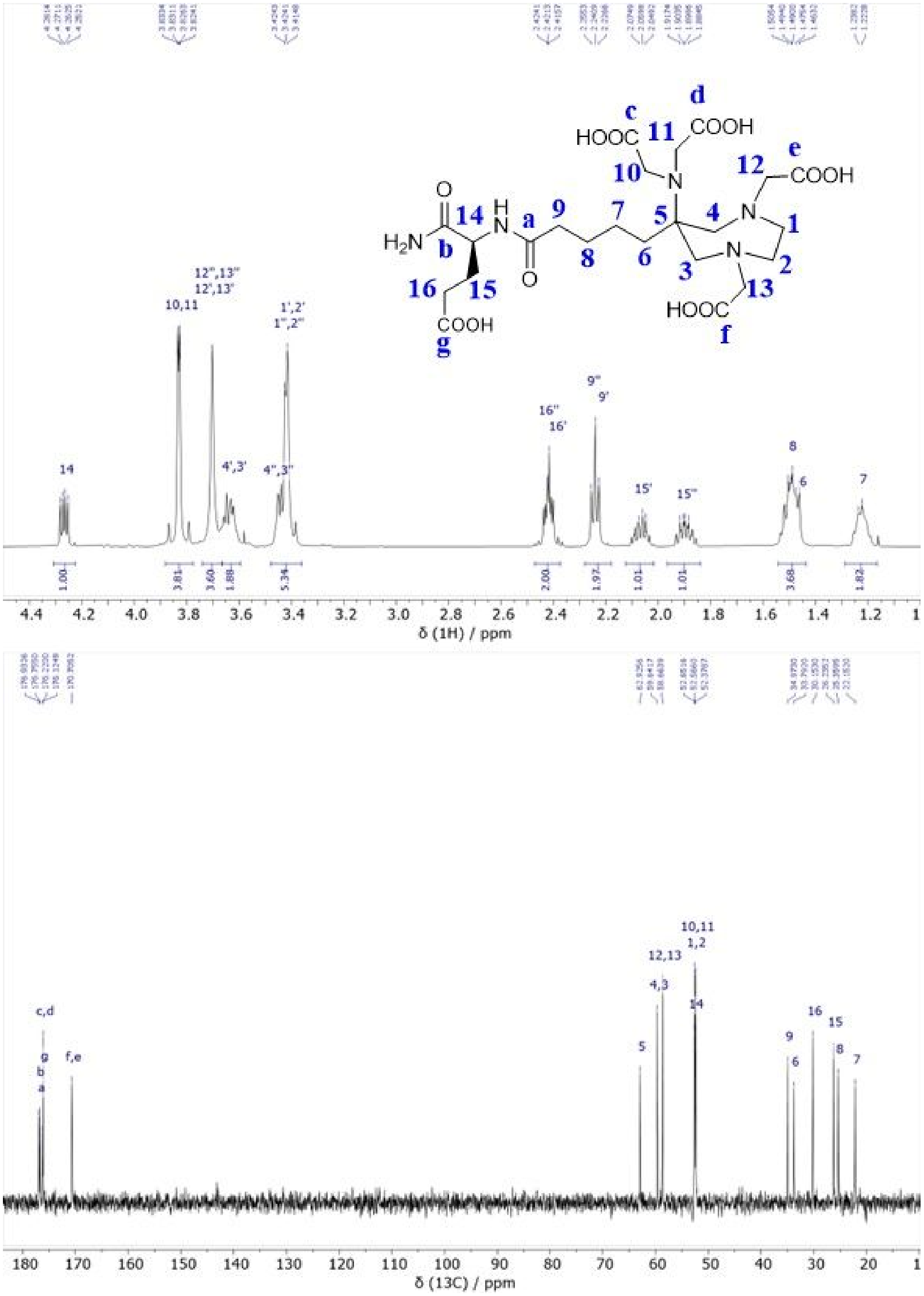

Figure S11. 1D ^{1}H and ^{13}C NMR spectra acquired for the AAZTA-Glu ligand at 300 K, 11.7 Tesla.

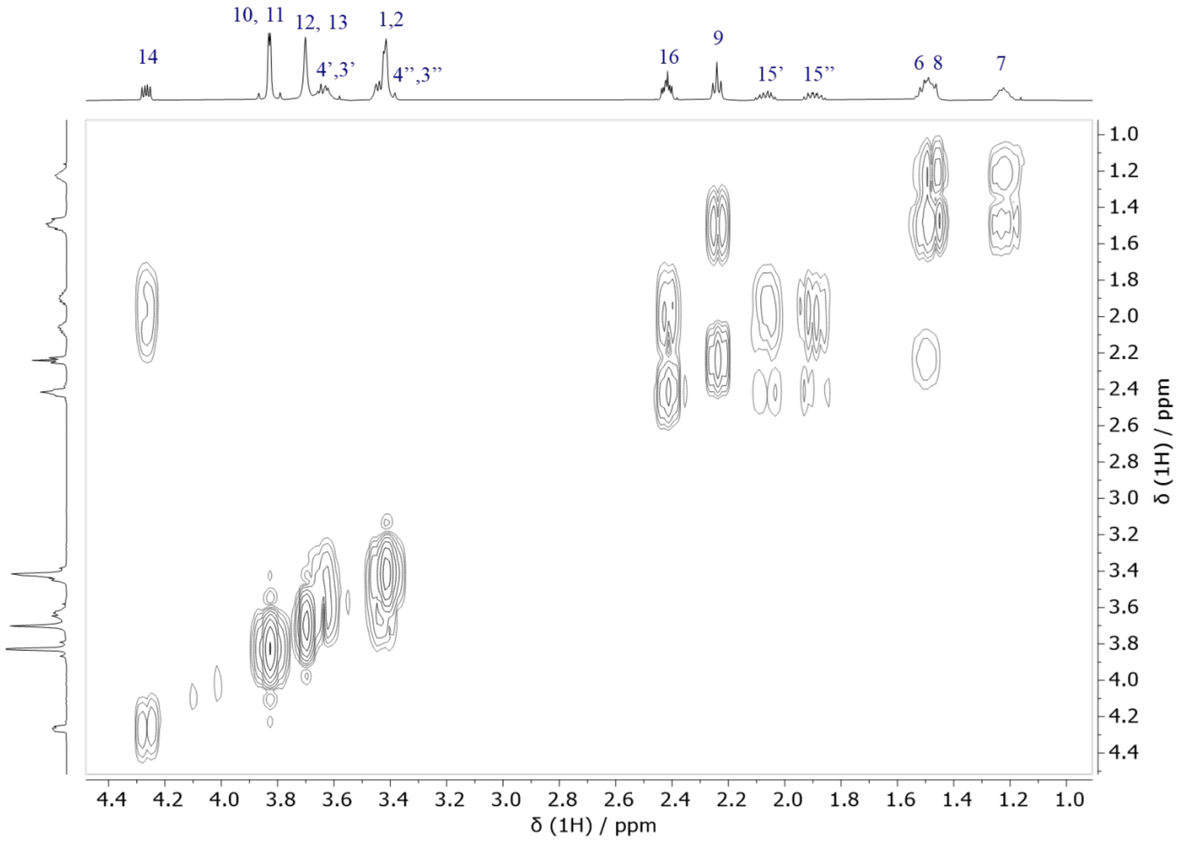

Figure S12. ^{1}H-^{1}H COSY 2D NMR spectrum acquired for the AAZTA-Glu ligand at 300 K, 11.7 Tesla.

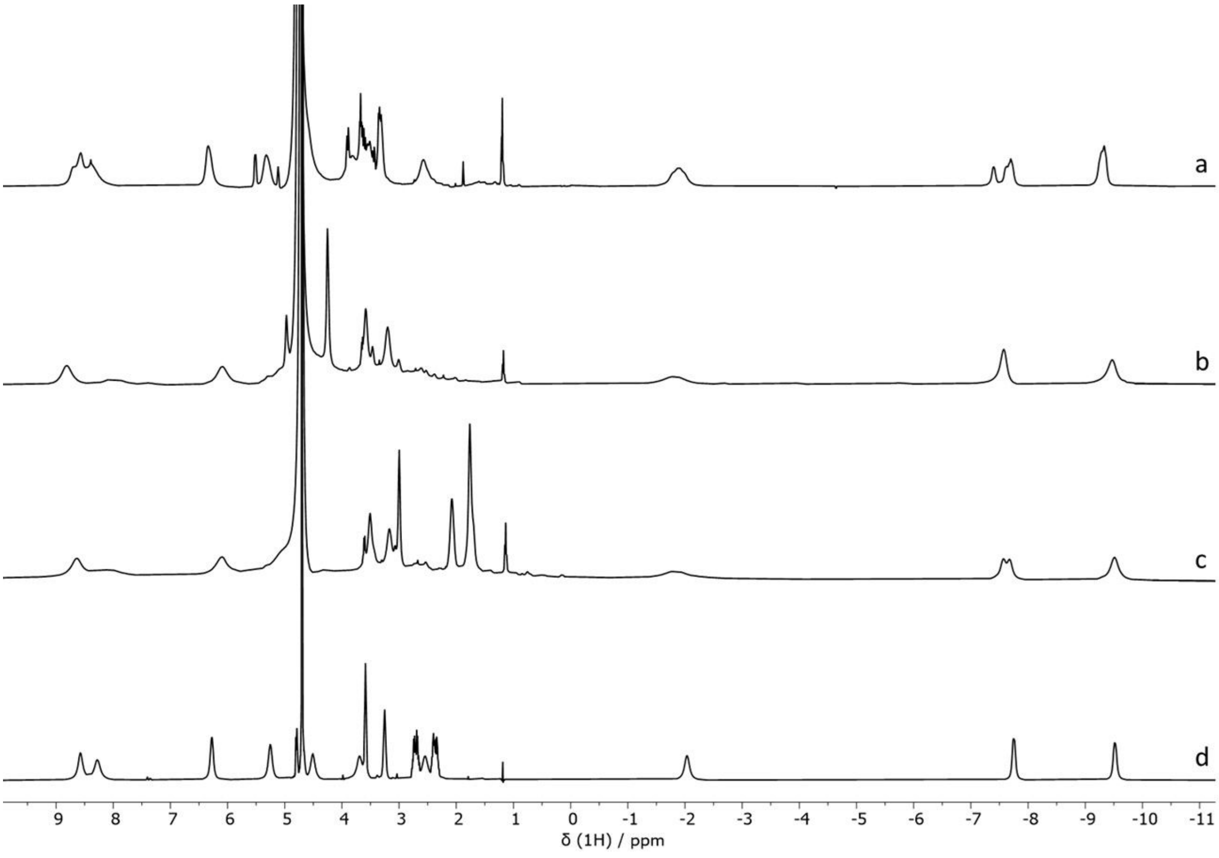

Figure S13. ^{1}H NMR spectra acquired at 11.7 T and 300 K of EuAAZTA-Cys (a), EuAAZTA-Ser (b), EuAAZTA-Lys (c) and EuAAZTA-Glu (d).

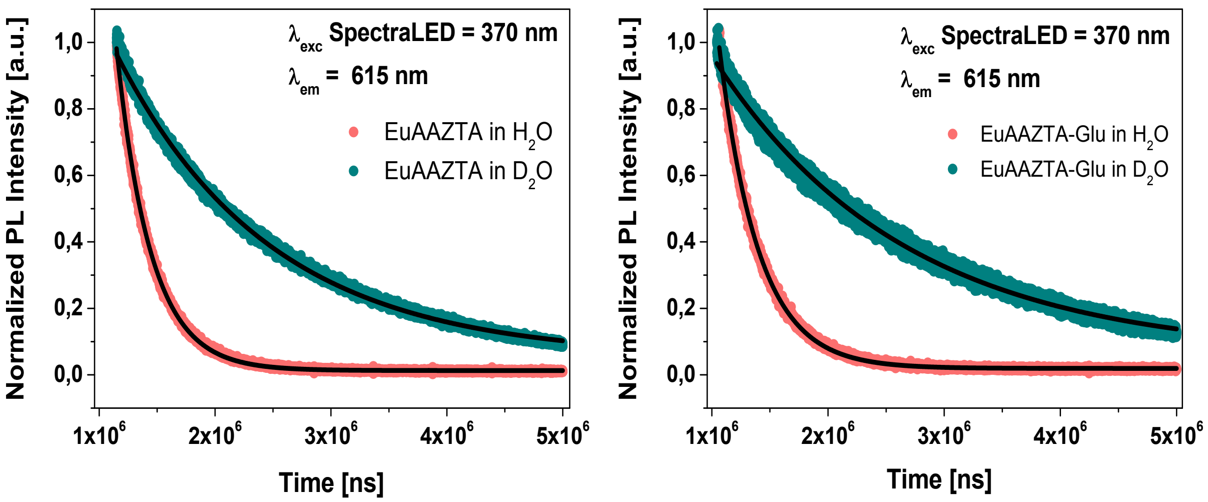

Figure S14. PL $^5D_0$-$^7F_2$ (615 nm) intensity decay profiles over time for the EuAAZTA (left) and EuAAZTA-Glu (right) complexes in H_2O (red) e D_2O (green), under excitation at 370 nm.

Table S1. Excited state lifetime values in H_2O (τ_{H_2O}) and in D_2O (τ_{D_2O}) obtained from the fitting procedure with a monoexponential decay function. The *q* parameter was calculated by applying the equation of ref. 1.

Complexes	τ_{H_2O} (ms)	τ_{D_2O} (ms)	*q*
**EuAAZTA**	0.319 ± 0.001	1.016 ± 0.003	2.33 ± 0.2
**EuAAZTA-Glu**	0.338 ± 0.001	1.354 ± 0.005	2.36 ± 0.2

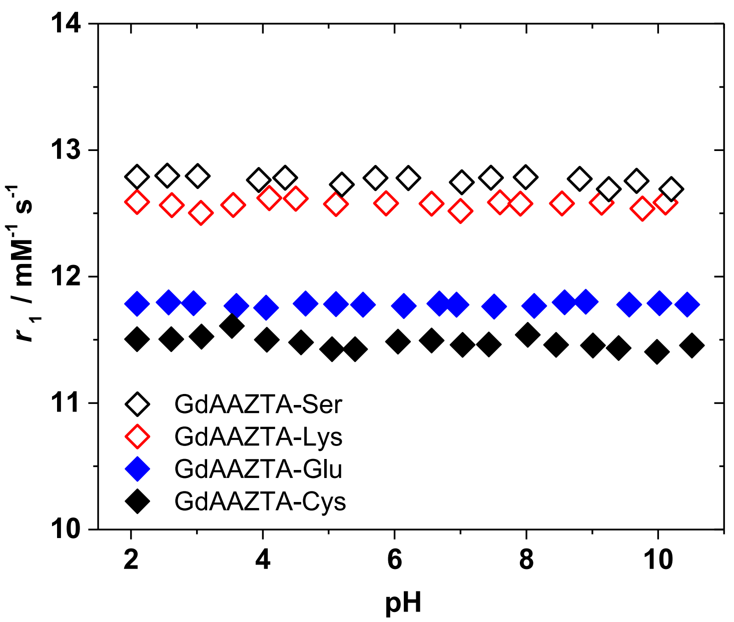

Figure S15. pH dependence of r_1 measured for aqueous solutions 2 mM of the GdAAZTA-aa complexes (aa = Cys, Ser, Lys and Glu), at 32 MHz and 298 K.

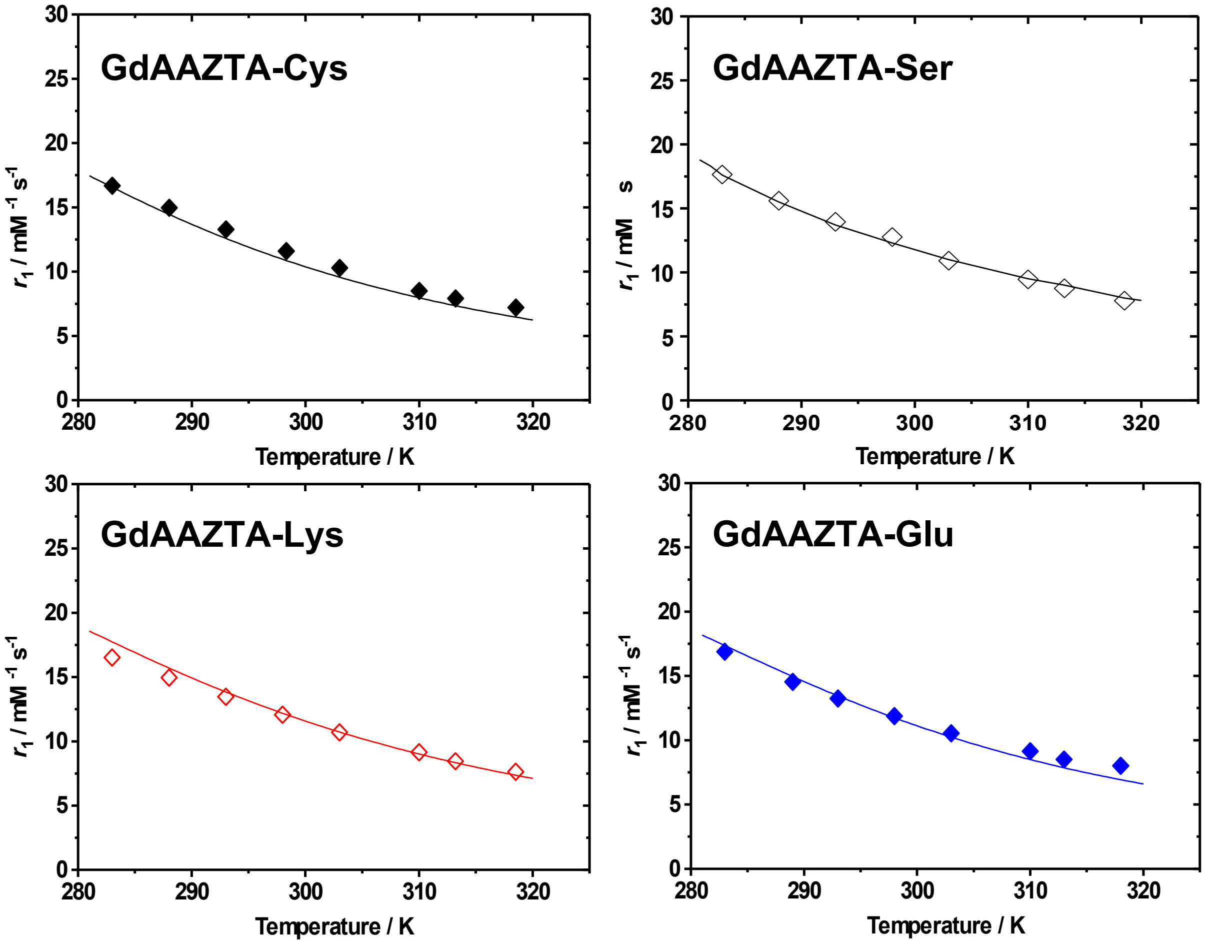

Figure S16. Temperature dependence of r_1 measured for aqueous solutions of the GdAAZTA-aa complexes ([Gd^{3+}] = 0.25 mM), at 32 MHz. The solid lines correspond to the fits of the data as described in the text.

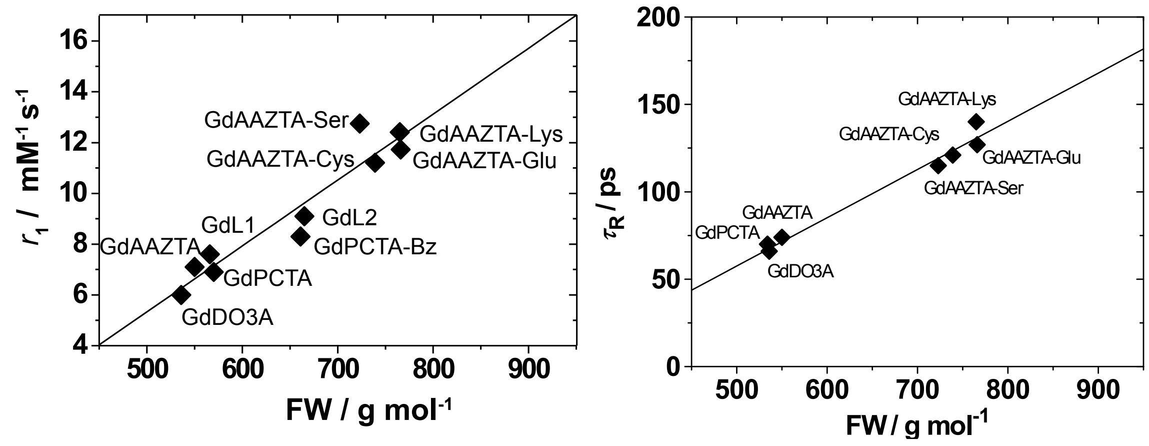

Figure S17. Left: plot of r_1 (0.5 T and 298 K) for aqueous solutions of GdAAZTA derivatives and other bi-hydrated Gd(III) chelates versus molecular weight (ref. 2). Right: Plot of the corresponding rotational correlation times as a function of molecular weight. L1 = 1,4-Bis(carboxymethyl)-6-[bis(carboxymethyl)]amino-6-hydroxymethylperhydro-1,4-diazepine and L2 = 1,4-Bis(carboxymethyl)-6-[bis(carboxymethyl)]amino-6-(2-oxa-3-oxo-5-carboxy)pentylperhydro-1,4-diazepine, at 32 MHz and 298 K.

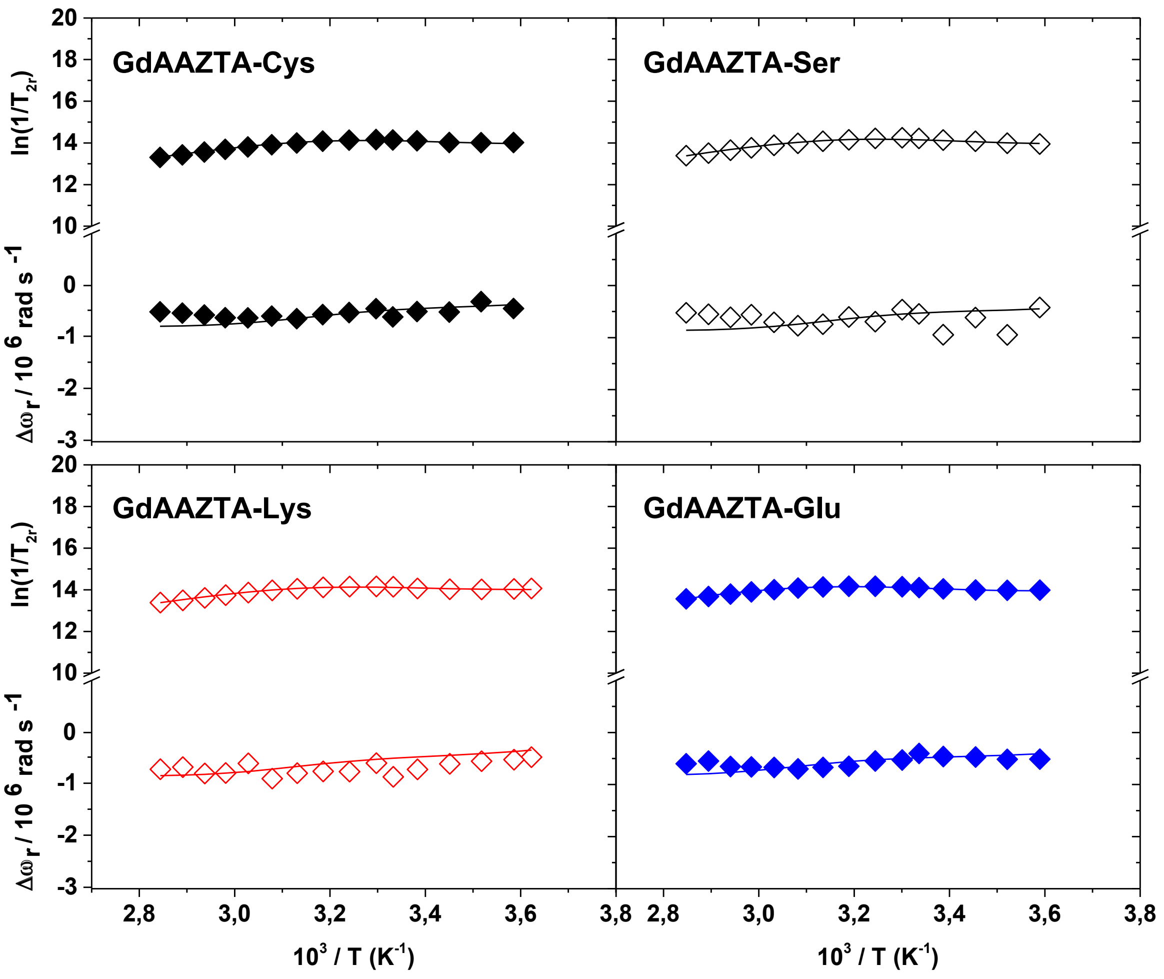

Figure S18. Reduced transverse ^{17}O relaxation rates and chemical shifts measured at 11.7 T for the GdAAZTA-aa complexes. The solid lines correspond to the fits of the data as described in the text.

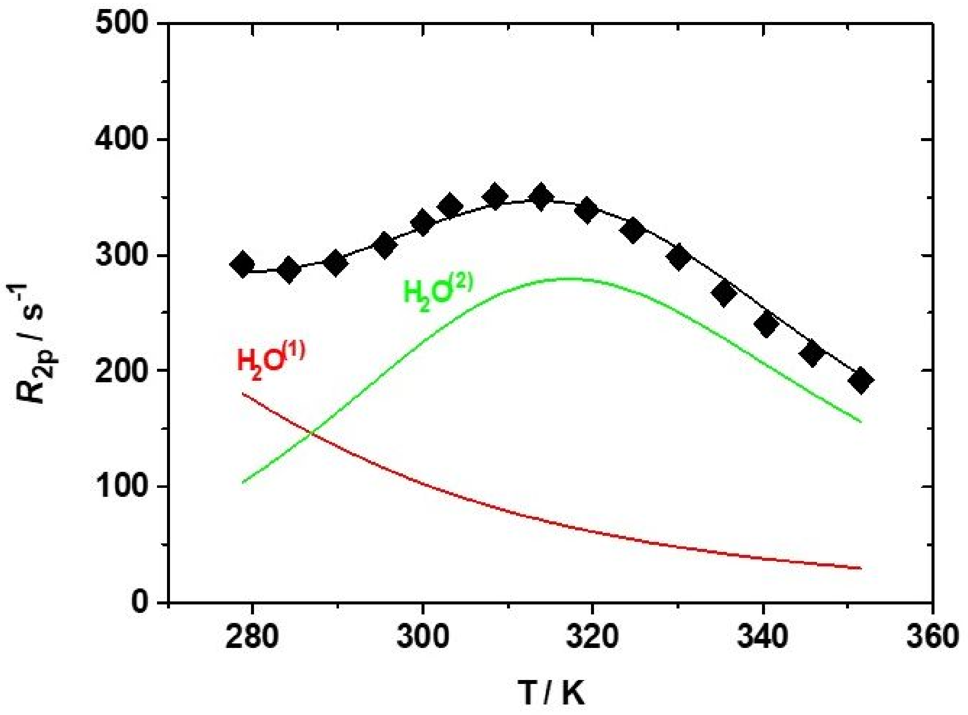

Figure S19: ^{17}O-R_2 profile of GdAAZTA-Glu. The distinct contribution of the two inner sphere water molecules is also reported.

Table S2. Parameters obtained from the simultaneous analysis of ^{17}O NMR and ^{1}H NMRD data acquired on the GdAAZTA-aa complexes.

	**GdAAZTA-Ser**	**GdAAZTA-Cys**	**GdAAZTA-Lys**	**GdAAZTA-Glu**	**GdAAZTA**^{b}
Δ^2 / 10^{19} s^{-2}	2.56	2.30	2.40	2.30	2.6
τ_V / ps	30.0	31.0	30.0	30.0	30.0
E_V/ kJ mol^{-1}	1.0^{a}	1.0^{a}	1.0^{a}	1.0^{a}	1.0^{a}
τ_{M1}/ ns	23.3	24.8	29.8	22.5	29
τ_{M2}/ ns	206	190	219.2	245	169
ΔH_{M1} / kJ mol^{-1}	18.0	18.5	20.0	19.0	20.0
ΔH_{M2} / kJ mol^{-1}	30.9	29.0	29.8	29.2	29.5
τ_R / ps	115	121	140	127	74
E_R / kJ mol^{-1}	23.0	23.0	24.0	23.9	20.0
$^{SS}\tau_R$ / ps	115	/	/	/	/
$^{SS}E_R$ / kJ mol^{-1}	23.1	/	/	/	/
$A_O/h_1/10^6$rad s^{-1}	-3.8	-3.7	-3.8	-3.9	-3.8
$A_O/h_2/10^6$rad s^{-1}	-3.9	-3.9	-3.8	-4.0	-3.9
q	2^{a}	2^{a}	2^{a}	2^{a}	2^{a}
r_{GdH} / Å	3.0^{a}	3.0^{a}	3.0^{a}	3.0^{a}	3.0^{a}
^{SS}q	1^{a}	/	/	/	/
^{SS}r / Å	3.5^{a}	/	/	/	/
a_{GdH} / Å	4.0^{a}	4.0^{a}	4.0^{a}	4.0^{a}	4.0^{a}
^{298}D / 10^5 cm^2 s^{-1}	2.24^{a}	2.24^{a}	2.24^{a}	2.24^{a}	2.24^{a}

[a]Parameters fixed during the fitting procedure. [b] data from Ref. 3.

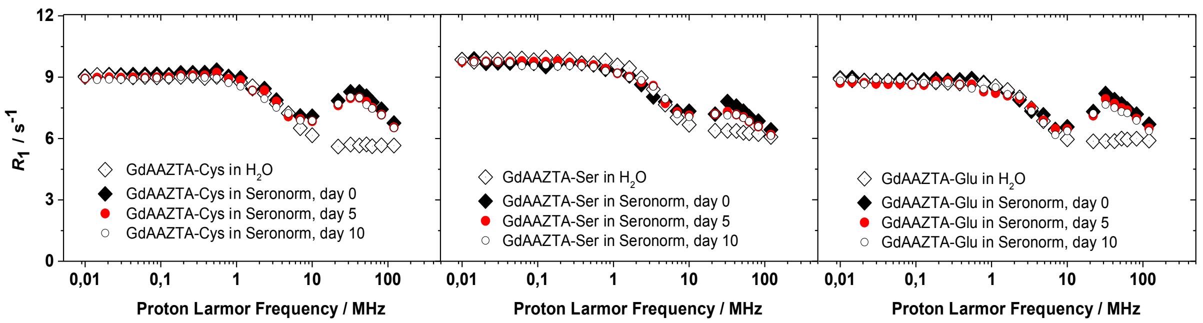

Figure S20. Comparison of R_1 values as a function of the magnetic field strength (0.01-120 MHz) measured on the GdAAZTA-aa complexes in pure water (◊), and in the presence of Seronorm® over the time. (298 K, pH 7.4, $[Gd^{3+}]$ = 0.5 mM).

Relaxation analysis

Paramagnetic complexes undergoing Brownian motions in solution are able to generate oscillating local magnetic fields, which induce relaxation of the nearby water molecules' nuclei. The water molecules interacting with such chelates can be classified into three categories: those of inner sphere (IS), directly coordinated to the metal ion and in exchange with the bulk water; of second sphere (SS), involved in hydrogen bonds with the polar groups of the complex; and of outer sphere (OS), diffusing close to the chelate. Each contributes to the relaxivity as follows:

$$r_1 = r_1^{IS} + r_1^{SS} + r_1^{OS} \quad (1)$$

where the inner-sphere water molecules contribute the most, thanks to their proximity to the metal center. The inner-sphere relaxivity (r_1^{IS}) describes a fast exchange dynamic between two pools of water molecules at different concentrations, namely the IS water molecules at lower concentration, and the bulk ones at higher concentration:

$$r_1^{IS} = \frac{1}{1000} x \frac{q}{55.55} x \frac{1}{T_{1M} + \tau_M} \quad (2)$$

r_1^{IS} depends on the hydration number (q), the residence time (τ_M) of metal-coordinated water molecules, and on their longitudinal relaxation time (T_{1M}). T_{1M} is described by the Solomon-Bloembergen-Morgan equations and depends on several molecular parameters, including the distance between the hydrogen atoms of the inner-sphere water molecules and the metal center ($r_{M\text{-}H}$), the magnetic fields strength (B_0) and the correlation time of the magnetic fluctuation (τ_c), defined as follows:

$$\frac{1}{\tau_c} = \frac{1}{\tau_R} + \frac{1}{\tau_M} + \frac{1}{T_{ie}} \quad (3)$$

where τ_R is the rotational correlation time, τ_M the water residency time and T_{ie} (i = 1, 2) the electronic relaxation times. Therefore, field-dependent relaxivity profiles measurements allow obtaining a detailed description of the molecular parameters controlling the relaxometric properties of paramagnetic chelates.

References

(1) Beeby, A.; Clarkson, I. M.; Dickins, R. S.; Faulkner, S.; Parker, D.; Royle, L.; de Sousa, A. S.; Williams, J. A. G.; Woods, M. Non-radiative deactivation of the excited states of europium, terbium and ytterbium complexes by proximate energy-matched OH, NH and CH oscillators: an improved luminescence method for establishing solution hydration states *Journal of the Chemical Society, Perkin Transactions 2*, **1999**, 3, 493-504.

(2) Gianolio, E.; Cabella, C.; Serra, S. C.; Valbusa, G.; Arena, F.; Maiocchi, A.; Miragoli, L.; Tedoldi, F.; Uggeri, F.; Visigalli, M.; Bardini P.; Aime, S. B25716/1: a novel albumin-binding Gd-AAZTA MRI contrast agent with improved properties in tumor imaging. *Journal of Biological Inorganic Chemistry*, **2014**, 19, 715-726.

(3) Lalli, D. Carniato, F. Tei, L. Platas-Iglesias, C. Botta, M. Surprising complexity of the $[Gd(AAZTA)(H_2O)_2]^-$ chelate revealed by NMR in the frequency and time domains. *Inorg. Chem.*, **2022**, 61, 496−506.
